# Recent Advances in Grayanane Diterpenes: Isolation, Structural Diversity, and Bioactivities from Ericaceae Family (2018–2024)

**DOI:** 10.3390/molecules29071649

**Published:** 2024-04-06

**Authors:** Sheng Liu, Lili Sun, Peng Zhang, Changshan Niu

**Affiliations:** 1 School of Pharmacy, Yantai University, Yantai 264005, China; liusheng87@126.com; 2 College of Pharmacy, University of Utah, Salt Lake City, UT 84108, USA; lili.sun1989@gmail.com (L.S.); u6024660@utah.edu (P.Z.)

**Keywords:** diterpene, grayanane, Ericaceae family, *Pieris*, *Rhododendron*, *Kalmia*, *Craibiodendron*, *Leucothoe*, pain assay, PTP1B, anti-inflammatory, analgesic, antifeedant

## Abstract

Diterpenes represent one of the most diverse and structurally complex families of natural products. Among the myriad of diterpenoids, grayanane diterpenes are particularly notable. These terpenes are characterized by their unique 5/7/6/5 tetracyclic system and are exclusive to the Ericaceae family of plants. Renowned for their complex structures and broad spectrum of bioactivities, grayanane diterpenes have become a primary focus in extensive phytochemical and pharmacological research. Recent studies, spanning from 2018 to January 2024, have reported a series of new grayanane diterpenes with unprecedented carbon skeletons. These compounds exhibit various biological properties, including analgesic, antifeedant, anti-inflammatory, and inhibition of protein tyrosine phosphatase 1B (PTP1B). This paper delves into the discovery of 193 newly identified grayanoids, representing 15 distinct carbon skeletons within the Ericaceae family. The study of grayanane diterpenes is not only a deep dive into the complexities of natural product chemistry but also an investigation into potential therapeutic applications. Their unique structures and diverse biological actions make them promising candidates for drug discovery and medicinal applications. The review encompasses their occurrence, distribution, structural features, and biological activities, providing invaluable insights for future pharmacological explorations and research.

## 1. Introduction

Diterpenes, a class of terpenoids consisting of four isoprene units, represent one of the most diverse and structurally complex families of natural products. As a prominent family of natural products, diterpenes are predominantly found in plants, where they play vital roles in various biological processes, from defense mechanisms against herbivores and pathogens to growth regulation [1]. The vast structural diversity and the array of bioactivities associated with diterpenes have made diterpenes a focal point of intense scientific research.

Among the myriad of diterpenes, grayanane diterpenes stand out as particularly noteworthy. These terpenes are distinguished by their unique and intricate 5/7/6/5 tetracyclic system and are exclusive to the Ericaceae family of plants [2,3,4]. The Ericaceae family, which encompasses about 4000 species spread across 126 genera, ranging from small herbs to large trees, is a rich source of terpenoids, including triterpenoids, meroterpenoids, and especially diterpenoids such as grayanane diterpenes [2,5]. Grayanane diterpenes, as characteristic secondary metabolites of the Ericaceae family, are prominently found in genera like *Pieris*, *Rhododendron*, *Kalmia*, *Craibiodendron*, and *Leucothoe*.

The structural complexity and diversity of grayanane diterpenes are notable, with over 400 compounds encompassing 25 carbon skeletons that have been isolated and identified from the Ericaceae family [2,6,7]. These compounds are recognized for their wide-ranging bioactivities, including analgesic [3,8], anti-inflammatory [9], antifeedant [10], and protein tyrosine phosphatase 1B (PTP1B) [11] inhibitory activities. Their unique chemical structures and significant biological activities have increasingly attracted the interest of organic synthesis chemists [12,13].

Despite several reviews that have covered aspects of grayanane diterpenoids, a comprehensive and in-depth overview of the developments and discoveries in this field, especially from 2018 to January 2024, has been lacking [2,6,7,14,15,16]. This review aims to fill that gap by focusing on the recent advancements made in the isolation, structural elucidation, and bioactivity studies of these diterpenes. Through a detailed examination of various species within the Ericaceae family, the paper presents a thorough overview of their occurrence, distribution, structural features, and biological activities. This approach offers valuable insights for ongoing pharmacological research and underscores the growing significance of grayanane diterpenes in the field of natural product chemistry.

## 2. Overview of Structural Diversity and Biological Activities of Grayanane Terpenes

**Figure 1:**
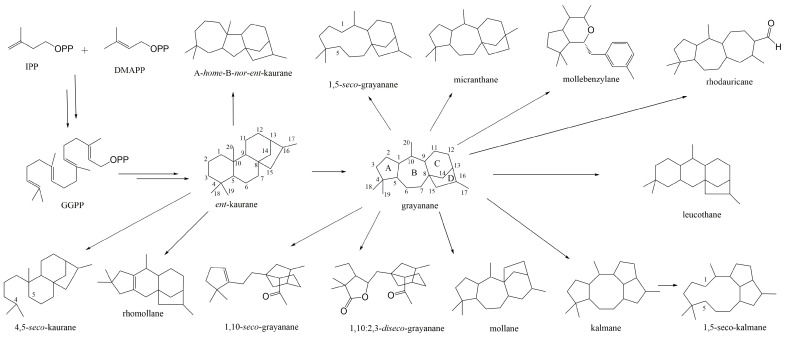
Representation of grayanane-related carbon skeletons. The core 5/7/6/5 skeleton of grayanane was labeled as rings A, B, C, and D.

After an exhaustive search of the PubMed, SciFinder, Scopus, and Google Scholar databases, utilizing the keywords “grayanane”, “diterpenes”, “diterpenoids”, and “Ericaceae family” from 2018 to January 2024, a remarkable total of 193 novel grayanane diterpenes were isolated and identified from the Ericaceae family plants. These discoveries predominantly came from the roots, leaves, or flowers of Pieris, Rhododendron, and Craibiodendron genus. These novel grayanane diterpenes are categorized into 15 distinct carbon skeletons, including *ent*-kaurane [17], 4,5-*seco*-kaurane [18], A-*home*-B-*nor*-*ent*-kaurane [17], grayanane [10], 1,5-*seco*-grayanane [19,20], 1,10-*seco*-grayanane [17], 1,10:2,3-*diseco*-grayanane [17,21], mollane [20,21], kalmane [19,20,22], 1,5-*seco*-kalmane [23], leucothane [18,21,23,24,25], rhomollane [23], micranthane [20,25], mollebenzylane [26], and rhodauricane [19], as illustrated in Figure 1.

Most of the literature research has focused on the bioactive potential of these compounds. A significant part of the studies is dedicated to analyzing their analgesic effects in vivo, particularly in mouse models. Various models have been employed for this purpose, including the acetic acid-induced writhing test and the capsaicin- and AITC-induced writhing test model [27]. Additionally, there have been studies on the antifeedant activity using *Plutella xylostella* [10], ion channel testing on Nav1.7 and KCNQ2 [10], anti-inflammatory properties [11], cytotoxicity [11], and PTP1B activity [11]. In the subsequent sections of the study, an in-depth exploration of the phytochemistry of these compounds is conducted. For detailed compound information, including the compounds’ original name, their occurrence, distribution, and publication references, please see Table 1. The bioactivities reported in the references were summarized in Table 2.

### 2.1. Normal Grayanane-Type Diterpenes (***1***–***97***)

**Figure 2:**
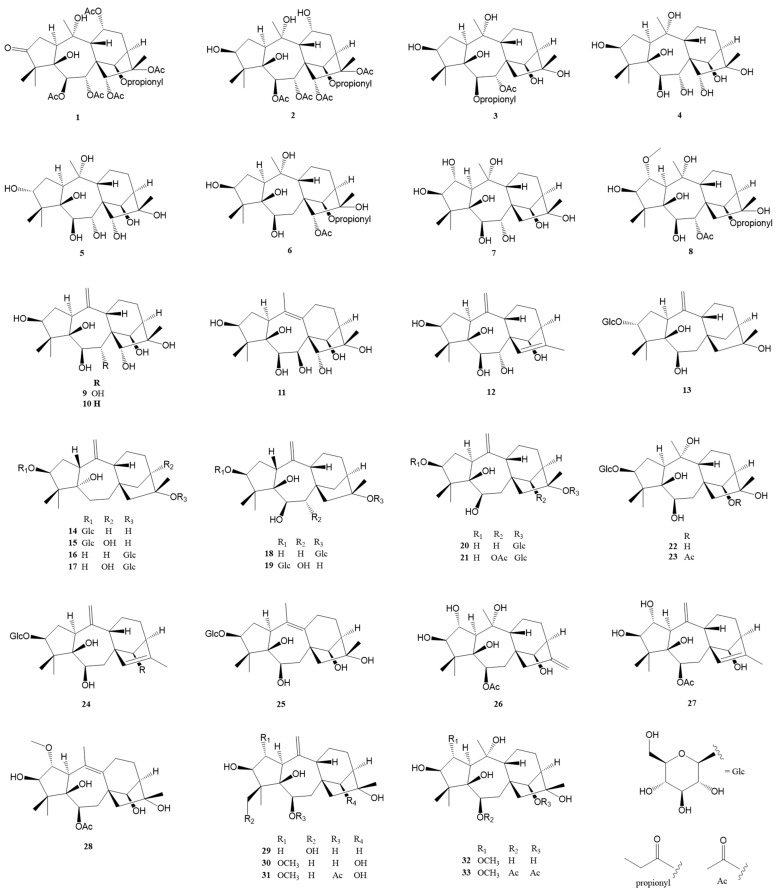
Structures of compounds **1**–**33**.

**Figure 3:**
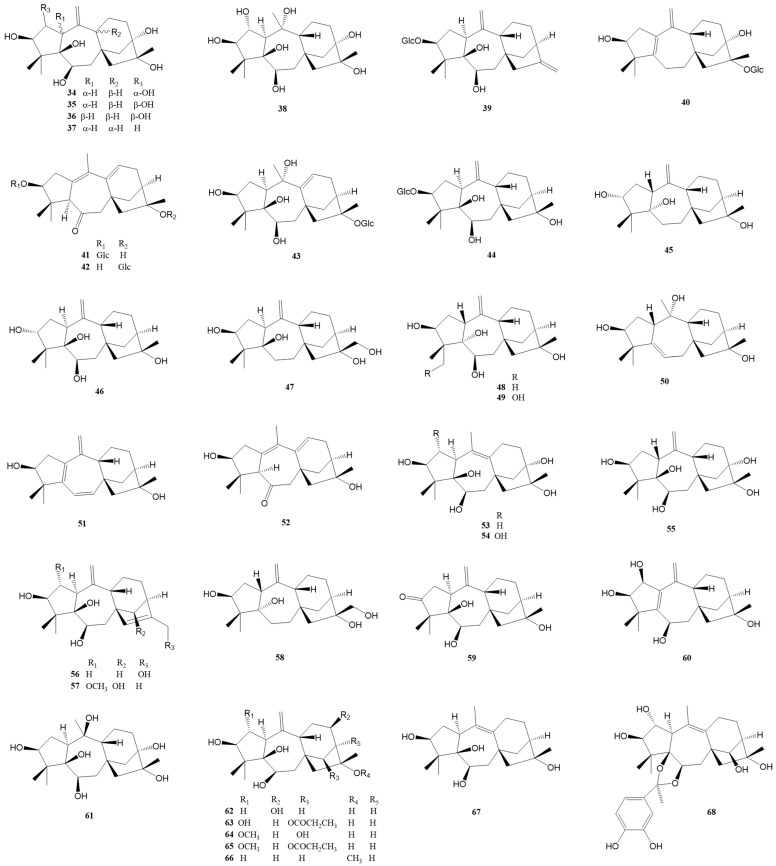
Structures of compounds **34**–**68**.

**Figure 4:**
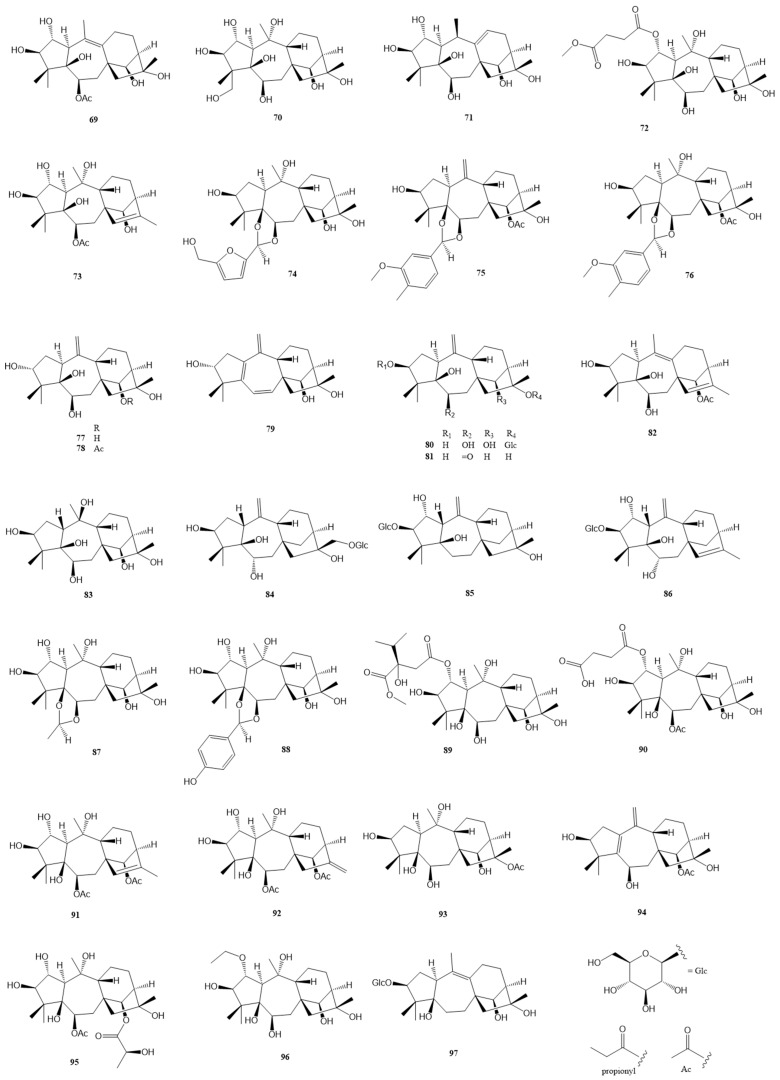
Structures of compounds **69**–**97**.

Normal grayanane diterpenes, a predominant class of diterpenes, have been the subject of extensive research, culminating in the discovery of 97 unique compounds. Characterized by their distinctive 5/7/6/5 tetracyclic framework, these compounds are depicted in Figure 2, Figure 3 and Figure 4 and elaborated upon in Table 1 and Table 2. This section meticulously explores the remarkable identification of these 97 novel grayanane diterpenes, each marked by a unique tetracyclic structure comprising four interconnected carbon rings. Notably, the grayanane diterpenes display a standard 5/7/6/5 configuration within their tetracyclic systems, a configuration that sets them apart from other diterpene structures. This divergence often translates into varied biological properties and potential applications, underscoring the significance of this discovery.

Pierisformosoids A-L (**1**–**12**) were isolated and identified from the roots of *Pieris formosa* [10]. Notably, compounds **1**, **2**, **4**–**5**, and **7**–**8** demonstrated significant analgesic activity in an acetic acid-induced writhing test in mice at a dosage of 5.0 mg/kg (i.p.), with compound **7** being five times more potent than positive control morphine. Compounds **1**, **4**, and **9** showed antifeedant activity against *Plutella xylostella* at 0.5 mg/mL. Compound **4** inhibited the KCNQ2 potassium channel by 38.3% at a concentration of 10 mM. Thirteen novel grayanane diterpenes (**13**–**25**) were isolated from the leaves of *R. micranthum*, and the structures were identified through extensive spectroscopic analysis and X-ray diffraction [11]. Compound **13** is notable as the first example of a 3α-oxygrayanane diterpenoid glucoside. Compounds **14**–**17** are the first examples of 5α-hydroxy-1-βH-grayanane diterpenoids, and compounds **16**–**18** and **20**–**21** represent the first grayanane glucosides with glucosylation at C-16. Compounds **14**, **15**, **19**–**22**, and **24**–**25** exhibited significant antinociceptive effects at 5 mg/kg, surpassing 50% inhibition using morphine as a positive control in the acetic acid-induced writhing test. Zhou et al. reported eight novel diterpenes compounds (**26**–**33**) from the leaves of *R. molle* [9]. Additionally, Zhu et al. identified seven new diterpenes (**34**–**39**) from the leaves and twigs of *R. decorum* [25], with compounds **34**, and **36**–**39** displaying significant antinociceptive activity at 10 mg/kg. Compound **38** was particularly potent, inhibiting 68.0% writhes at a dose of 0.8 mg/kg.

Five analgesic grayanane diterpene glucosides, **40** [24] and **41**–**44** [17], were isolated and illustrated from leaves of *R. auriculatum* and *R. micranthum*, respectively. At a dose of 1.0 mg/kg, compound **40** displayed notable analgesic activity with the acetic acid-induced writhing test. Compound **43** significantly reduced the number of writhes with an inhibition rate of over 50% at the same dosage. Compounds **45**–**55**, isolated by Sun et al. from the leaves of *R. auriculatum*, and their structures were defined via extensive spectroscopic data analysis and X-ray diffraction analysis [28]. Compound **45** represents the first example of a 3α,5α-dihydroxy-1-βH-grayanane diterpenoid, while **49** and **50** are the first examples of 19-hydroxygrayanane and grayan-5(6)-ene diterpenoids, respectively. Compounds **45**–**55** all showed significant analgesic activities at 5.0 mg/kg in an acetic acid-induced writhing test with an inhibition rate over 50%. From a leaf extract of *P. japonica*, twelve novel antinociceptive grayanane diterpenoids, **56**–**67**, were isolated and determined by spectroscopic methods as well as X-ray diffraction analysis [29]. Compound **56** represents the first example of a 17-hydroxygrayan-15(16)-ene diterpenoid and exhibited potent antinociceptive effects with writhe inhibition rates of 56.3% and 64.8% at doses of 0.04 and 0.2 mg/kg, respectively, with effects comparable to the positive control morphine in the HOAc-induced writhing test in mice. 

Li et al. reported six novel grayanane diterpenes (**68**–**73**) from the flowers of *R. molle* [23], with compound **71** inhibiting 46.0% of acetic acid-induced writhes at a dose of 2.0 mg/kg. Three 1,3-dioxolane conjugates of grayanane diterpenoids (**74**–**76**) with 5-hydroxymethylfurfural and vanillin, respectively, were isolated from the flowers of *R. dauricum* [19]. The structures were determined by spectroscopic methods and confirmed by X-ray diffraction analysis. At a lower dose of 0.04 mg/kg, **75** and **76** exhibited more potent activity than morphine in efficacy with inhibition rates of 62.8% and 53.2%, respectively. In chemical investigation of the flowers of *R. dauricum*, seven highly oxygenated grayanane diterpenes (**77**–**83**) were discovered [30], with compound **79** being a notable conjugated grayan-1(5),6(7),9(10)-triene diterpenoid. Among compounds **84**–**86,** purified from the leaves of *C. yunnanense* [31], **84** and **85** displayed significant anti-inflammatory activity, particularly inhibiting IL-6 release in lipopolysaccharide (LPS)-induced RAW264.7 cells. Zheng et al. identified six new diterpenes (**87**–**92**) from the flowers of *R. molle* as potent analgesics [32]. Notably, compound **92** demonstrated remarkable activity, remaining effective even at the dose of 0.04 mg/kg in vivo pain assay screenings. Chai et al. discovered compounds **93** and **94** from the roots of *R. micranthum* [22], both showing strong antinociceptive effects at doses of 0.1 mg/kg and 0.8 mg/kg, respectively. More recently, three additional minor grayanane diterpenes (**95**–**97**) were isolated and elucidated from the leaves of *C. yunnanense* [33].

### 2.2. Epoxy-Grayanane (***98***–***132***)- and Seco-Grayanane (***133***–***142***)-Type Diterpenes

**Figure 5:**
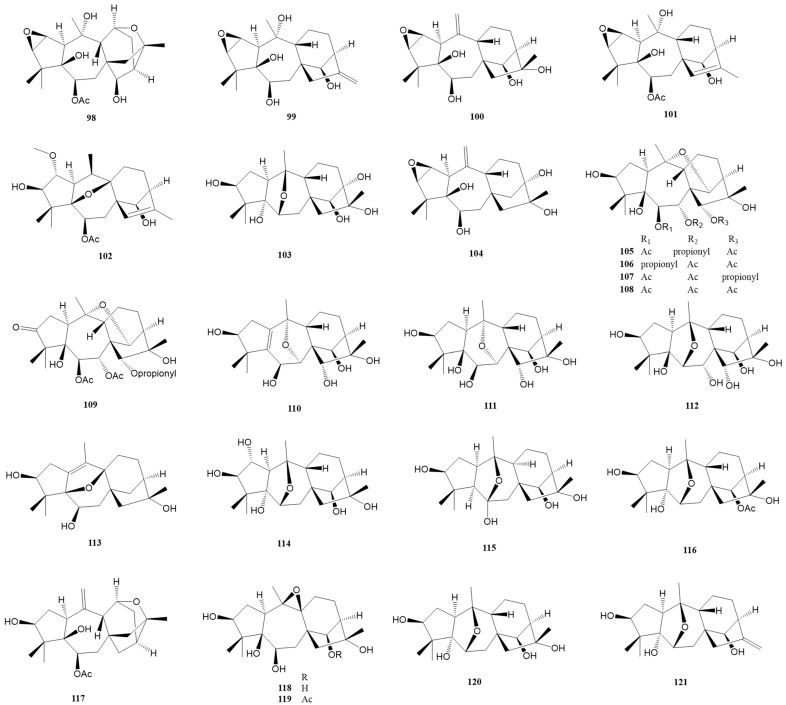
Structures of compounds **98**–**121**.

**Figure 6:**
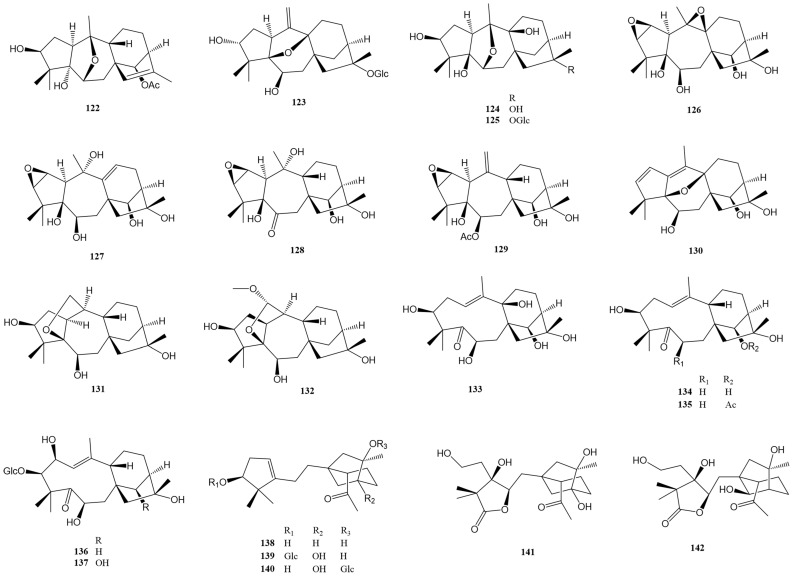
Structures of compounds **122**–**142**.

Epoxy-grayanane diterpenes represent a unique subset within the larger grayanane family, distinguished primarily by their epoxy group moiety. These compounds, numbering thirty-five in total, are defined by the inclusion of one or two epoxy groups in their structure. The positioning of these epoxy groups varies, occurring between different sets of carbon atoms. This variation leads to a range of configurations, such as C2-C3, C6-C10, C7-C10, C5-C9, C9-C10, C5-C20, C11-C16, and even combinations like C2-C3 with C9-C10, and C2-C3 with C11-C16. These configurations are detailed in Figure 5 and Figure 6 and Table 1. 

In addition, there is a category known as *seco*-grayanane diterpenes, of which eight varieties have been identified. These compounds are marked by a distinct feature: a structural ring opening, which results in different types, including 1,5-*seco*-grayanane, 1,10-*seco*-grayanane, and 1,10:2,3-*diseco*-grayanane. These are illustrated in Figure 6 and also listed in Table S1. The diversity in the structure of these diterpenes, particularly the placement and number of epoxy groups, contributes to their unique chemical properties and potential applications. The existence of both epoxy-grayanane and *seco*-grayanane diterpenes within the grayanane family highlights the complexity and variety inherent in natural compounds. The detailed categorization and identification of these compounds, as shown in the figures and tables, provide a valuable framework for further research and understanding of their characteristics and uses. 

Zhou et al. reported the isolation of five epoxy-grayanane diterpenes (**98**–**102**) from *R. molle* [9]. Notably, compound **98** represents the first example of a 2,3:11,16-diepoxy grayanane diterpenoid, showcasing a unique cis/trans/cis/cis/trans-fused 3/5/7/6/5/5 hexacyclic ring system with a 7,13-dioxahexacyclo-[10.3.3.0^1,11^.0^4,9^.0^6,8^.0^14,17^]octadecane scaffold. The structure was confirmed through X-ray diffraction analysis. Compound **100** exhibited significant anti-inflammatory activity in LPS-stimulated RAW264.7 mouse macrophages with an IC_50_ at 35.4 ± 3.9 μM. Two additional epoxy-grayanane diterpenes (**103**–**104**) were reported with a hydroxy group replaced at C-13 [25]. At 10.0 mg/kg, compound **103** displayed a mild antinociceptive effect. Furthermore, diverse epoxy-grayanane diterpenes (**105**–**112**) with analgesic activity were isolated from the roots of *P. formosa* [34]. Compounds **105**–**109** represent the first example of natural grayanane diterpenoids possessing a 10,14-epoxy group, while compounds **110**–**111** are the first example of grayanane diterpenoids possessing a 7,10-epoxy group. Compounds **105**–**107** and **109**–**112** showed significant analgesic activity at a dose of 5.0 mg/kg (i.p.) in the acetic acid-induced writhing test, with ibuprofen and morphine as the positive controls. 

Compound **113**, the second example of a 5β,9β-epoxygrayan-1(10)-ene diterpenoid, exhibited noticeable antinociceptive activity at 5.0 mg/kg in the acetic acid-induced writhing test in mice [29]. Three 6,10-epoxy grayanane diterpenes (**114** [23] and **115**–**116** [20]) were reported from *R. molle* and *R. micranthum*, respectively. Compound **115** represents the first example of a 5αH,9αH-grayanane diterpenoid and a 6-hydroxy-6,10-epoxy grayanane diterpenoid. Compounds **117**–**122** with diverse epoxy groups were isolated from the flowers of *R. dauricum* [30]. Compound **117** is the first example of an 11,16-epoxygrayan-6-one diterpenoid, while compounds **118** and **119** are the first examples of 9β,10β-epoxy grayanane diterpenoids. All these compounds (**117**–**122**) displayed significant analgesic activity in the acetic acid-induced writhing test in mice at 5.0 mg/kg, with inhibition rates over 50%. Compounds **117** and **122** were particularly potent, showing notable analgesic activity even at a lower dose of 0.2 mg/kg, with inhibition rates of 54.4% and 55.2%, respectively. Li et al. reported three undescribed epoxy-grayanane diterpenes (**123**–**125**) from *C. yunnanense*, with compound **125** notably inhibiting pro-inflammatory cytokines IL-6 at 10 μg/mL [31]. Six highly functionalized epoxy diterpenes (**126**–**131**) were elucidated by Zheng et al. from the flowers of *R. molle* [32]. Compounds **126**, **127**, and **130** are the first representatives of 2β,3β:9β,10β-diepoxygrayanane, 2,3-epoxygrayan-9(11)-ene, and 5,9-epoxygrayan-1(10),2(3)-diene diterpenoids, respectively. Compound **131** exhibited an inhibition rate of 51.4%, showing a more potent analgesic effect than morphine at a lower dose of 0.2 mg/kg in the acetic acid-induced writhing model. Compound **132** is another grayanane diterpene featuring a 5,20-epoxy group [28].

Compounds **133** [20] and **134**–**135** [19], displaying a 1,5-*seco*-grayanane carbon skeleton, were identified from *R. micranthum* and *R. dauricum*, respectively. Significantly, compounds **134** and **135** represent the first examples of 6-deoxy-1,5-*seco*-grayanane diterpenoids. Compounds **136**–**137** are distinguished as the first 1,5-*seco*-grayanane diterpenoid glucosides. Interestingly, these compounds exhibited only 17 carbon resonances instead of 26 carbons in their ^13^C NMR spectra. Their structures were conclusively determined by single-crystal X-ray diffraction [21]. The rare 1,10-*seco*-grayanane diterpenes, compounds **138**–**140**, were identified from the extracts of the leaves of *R. auriculatum*. Their structures were elucidated using NMR and ECD data analysis and were further confirmed by X-ray diffraction [24]. Additionally, two 1,10:2,3-*diseco*-grayanane diterpenes, compounds **141** [24] and **142** [21], were successfully reported. The primary difference between these two compounds is the absence of the OH-13 group in compound **142**.

### 2.3. Grayanane Dimers-Type Diterpenes (***143***–***149***)

**Figure 7:**
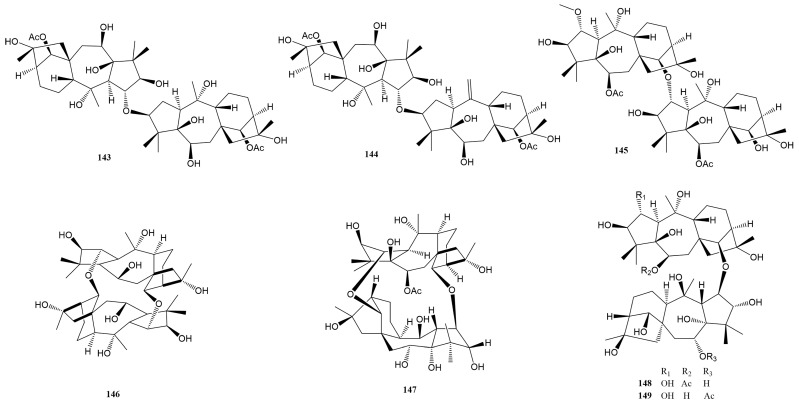
Structures of compounds **143**–**149**.

In the referenced scientific literature, there is a notable report detailing the discovery of seven unique grayanane dimer diterpenes. This significant finding is visually documented in Figure 7 and comprehensively listed in Table 1. These dimer compounds, which represent a unique and complex class of natural products, are characterized by their distinctive structural formation. Specifically, they are formed through the connection of two grayanane monomer units. This connection is achieved via one or two ether bonds, a type of chemical bond that involves an oxygen atom linked to two alkyl or aryl groups.

Two new dimeric diterpenes (**143** and **144**) were characterized from the fruits of *R. pumilum*, representing the first examples of dimeric grayanane diterpenes with a 3-O-2′ linkage from the Ericaceae family [35]. Another novel dimeric diterpene **145** [9] was identified from the leaves of *R. molle* but with a 13-O-2′ linkage. Compound **146** is a unique dimeric grayanoid, isolated from the flowers of *R. molle* [23], containing a novel 14-membered heterocyclic ring with a C_2_ symmetry axis. More recently, Huang et al. reported three new dimers, **147**–**149**, also from the flowers of *R. molle* [27]. The structures were determined by comprehensive spectroscopic data analysis, ^13^C NMR calculation with DP4+ analysis, and single-crystal X-ray diffraction analysis [27]. Of particular interest is compound **147**, a caged dimeric grayanane diterpenoid linked through two oxygen bridges of C-2−O−C-14′ and C-14−O−C-2′, featuring a unique 1,8-dioxacyclotetradecane motif. At a dose of 5.0 mg/kg, compounds **147**–**149** showed significant analgesic effects, with writhe inhibition rates exceeding 50% in the acetic acid-induced writhing test. Even at a lower dose of 1.0 mg/kg, compound **148** maintained an inhibition rate of 57.3%. Furthermore, in capsaicin- and AITC-induced pain models, compound **148** effectively reduced the nociceptive responses at a dose of 5.0 mg/kg, indicating its potential as a dual antagonist of TRPV1 and TRPA1.

### 2.4. Leucothane-Type Diterpenes (***150***–***163***)

**Figure 8:**
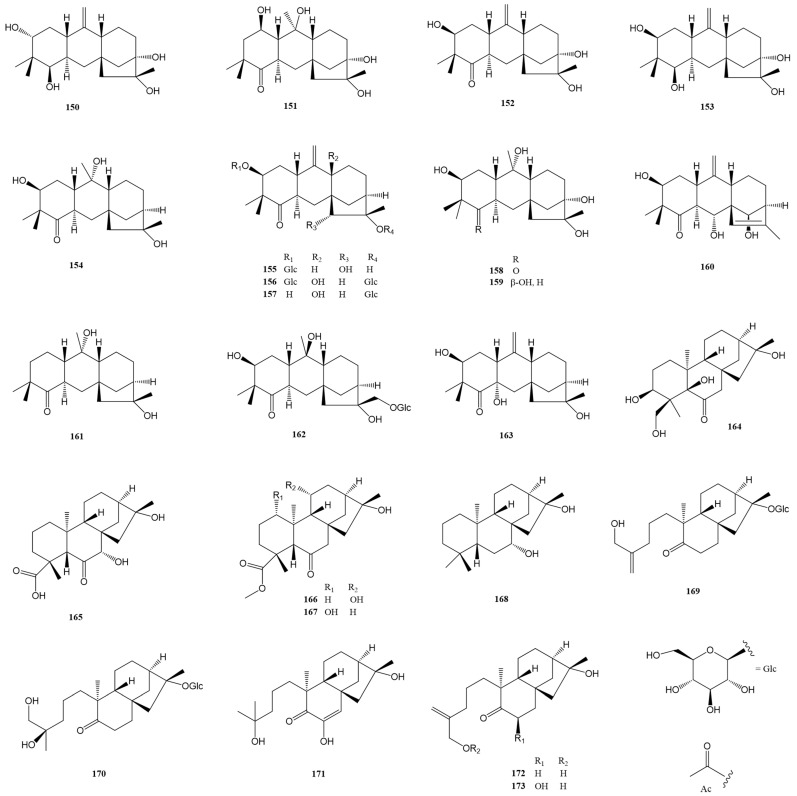
Structures of compounds **150**–**173**.

Leucothane-type diterpenes represent a fascinating subset within the broader category of grayanane-type diterpenes, known for their unique biosynthetic relationships. These compounds are distinguished by their distinct structural framework, which features a 6/6/6/5 fused tetracyclic ring system. Over the past five years, there has been notable progress in the identification and characterization of these compounds. Fourteen new leucothane-type diterpenes have been discovered and reported, marking a significant advancement in the study of naturally occurring diterpenes. Details are shown in Figure 8 and Table 1 and Table 2.

Three new leucothane-type diterpenes (**150**–**152**) were isolated from the leaves and twigs of *R. decorum* [25]. The structure of compound **150** was confirmed by X-ray crystallography. In the acetic acid-induced writhing test, compound **150** showed a significant effect at a dose of 10.0 mg/kg. Sun et al. reported five new leucothane-type terpenes (**153**–**154** [24] and **155**–**157** [17]) from *R. auriculatum* and *R. micranthum*, respectively. Compounds **155**–**157** represent the first examples of 15α-hydroxy-leucothane diterpenoids, leucothane diterpene diglucosides, and 9β-hydroxy-leucothane diterpenoids, respectively. These compounds (**153**–**157**) all displayed potent analgesic activity in the acetic acid-induced writhing test. Four additional leucothane-type diterpenes (**158**–**159** [23] and **160**–**161** [18]) were elucidated from *R. molle* and *P. formosa*, respectively. Compounds **159** and **160** demonstrated weak analgesic activity in the acetic acid-induced writhing test at 20.0 mg/kg and 5.0 mg/kg, respectively. In an antifeedant assay against *Plutella xylostella* larvae, compound **161** showed an inhibition effect with a ratio of 52.5% at a dose of 0.5 mg/mL. Lastly, two new leucothane-type diterpenes (**162**–**163**) were isolated and identified from *P. japonica* [21]. The structure of **163** was definitively confirmed through X-ray diffraction analysis. Notably, compound **162** exhibited strong analgesic activity with writhe inhibition over 50% at 5.0 mg/kg (i.p.).

### 2.5. Ent-Kaurane (***164***–***168***)- and Seco-Ent-Kaurane (***169***–***173***)-Type Diterpenes

*Ent*-kaurane-type diterpenes hold a crucial position in the biosynthesis of grayanane diterpenes, serving as bio-precursors in the intricate chemical pathways leading to the formation of grayanane structures. This role highlights the importance of understanding *ent*-kaurane-type diterpenes, not only for their inherent chemical properties but also for their contribution to the biosynthesis of other significant diterpenes. In the past five years, there has been a notable advancement in the research and identification of these compounds. Specifically, five *ent*-kaurane-type diterpenes and five 4,5-*seco*-*ent*-kaurane-type diterpenes have been successfully identified and reported. The 4,5-*seco*-*ent*-kaurane type represents a variation of the *ent*-kaurane structure, characterized by a unique opening in the ring structure, specifically between the 4th and 5th carbon atoms, which significantly alters their chemical and potentially biological properties. These discoveries are meticulously detailed in Figure 8 and Table 1 and Table 2.

Sun et al. and Niu et al. successfully reported the new *ent*-kaurane-type diterpenes **164** [24] and **165**–**168** [18] from the leaves of *R. auriculatum* and the roots of *P. formosa*, respectively. A detailed analysis of the spectroscopic methods and ECD calculations illustrated the structures of these compounds. At 5.0 mg/kg, compounds **164** and **166** displayed weak analgesic activity in the acetic acid-induced writhing test. Compound **167** showed antifeedant activity against *Plutella xylostella* larvae with an inhibition ratio of 27.1% at 0.5 mg/mL. Additionally, five 4,5-*seco*-*ent*-kaurane-type diterpenes (**169**–**170** [17], **171** [18], and **172**–**173** [21]) were successfully reported. Compounds **169**–**170**, identified as diterpene glucosides at C-17, demonstrated potent analgesic effects at a 1.0 mg/kg dose in an acetic acid-induced writhing test.

### 2.6. Kalmane (***174***–***179***)- and Seco-Kalmane (***180***)-Type Diterpenes

**Figure 9:**
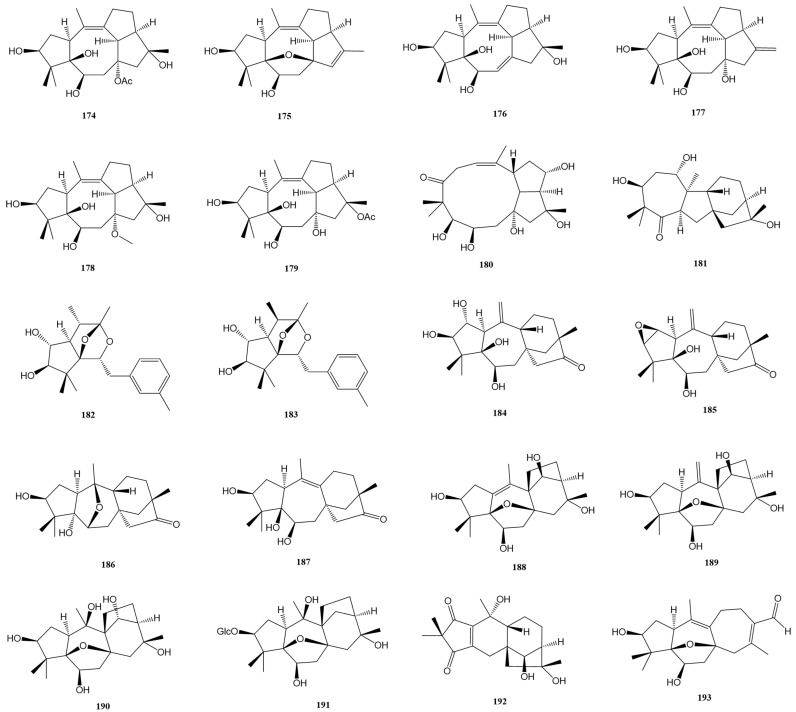
Structures of compounds **174**–**193**.

Kalmane-type diterpenes stand out as a rare and intriguing class of terpenes that originate from the grayanane type. They are particularly renowned for their distinctive structural feature: a 5/8/5/5 fused tetracyclic ring system. This structure is not commonly found in terpenes, making the kalmane type a subject of significant interest in the study of natural products and organic chemistry. In the last five years, there has been substantial progress in identifying and reporting new kalmane-type diterpenes. Specifically, six kalmane-type diterpenes, **174** [20], **175**–**178** [36], **179** [22], and one 1,5-*seco*-kalmane-type **180** [23] have been reported, as illustrated in Figure 9 and Table 1 and Table 2. Compound **175** is particularly noteworthy as it represents the first 5,8- epoxykalmane diterpenoid and the first kalm-15(16)-ene diterpenoid. Compounds **176**–**178** are the first examples of kalm-7(8)-ene, kalm-16(17)-ene, and 8α-methoxykalmane diterpenoids, respectively. The structures of compounds **174**–**176** and **178** were undoubtedly elucidated via X-ray diffraction analysis. Regarding bioactivity, diterpenes **175**–**178** exhibited significant analgesic effects in an acetic acid-induced writhing test. Remarkably, compound **177** showed even more potent activity at a very low dose of 0.04 mg/kg.

### 2.7. Other Grayanane-Related Diterpenes (***181***–***193***)

This section focuses on a fascinating group of grayanane-related diterpenes characterized by their rare and rearranged carbon skeletons. These compounds, derived from various genera, showcase the remarkable diversity and complexity found in natural products, particularly in the realm of terpenoid chemistry. These compounds span a range of structural variations, including A-*home*-B-*nor*-*ent*-kaurane **181** [24], mollebenzylanes **182**–**183** [26], micranthanes **184**–**187** [20,25], mollanes **188**–**191** [20,21], rhomollane **192** [23], and rhodaruricane **193** [19], as shown in Figure 9 and Table 1 and Table 2.

Compounds **182** and **183** are particularly notable for their unprecedented diterpene carbon skeleton, featuring a unique 9-benzyl-8,10-dioxatricyclo[5.2.1.0^1,5^]decane core. The absolute structure of **182** was unambiguously determined via X-ray diffraction analysis of its p-bromobenzoate ester. Compound **186** is the first 6,10-epoxymicranthane, while compounds **188** and **189** represent the first examples of 14β- hydroxymollane diterpenoids. Compound **191** is distinguished as the first mollane diterpene glucoside. Rhomollane **192** possesses an unprecedented 5/6/6/5 tetracyclic ring system (B-*nor* grayanane), incorporating a cyclopentene-1,3-dione scaffold. Its structure was undoubtedly solved by Mosher’s method and X-ray diffraction of its Mosher ester. Rhodaruricane **193** features a unique 5/6/5/7 tetracyclic ring system with a 16-oxa-tetracyclo[11.2.1.0^1,5^.0^7,13^]hexadecane core. Quantum chemical calculations, including 13C NMR-DP4+ analysis ECD calculations, and single-crystal X-ray diffraction analysis, elucidated the absolute structure of **193**. In terms of biological activity, compounds **181**, **184**, and **185** showed significant antinociceptive activity in the acetic acid-induced writhing test at 5.0 mg/kg, with **184** maintaining significant activity even at 1.0 mg/kg. Compounds **182** and **183** exhibited moderate PTP1B inhibitory activities with IC_50_ values of 22.99 ± 0.43 and 32.24 ± 0.74 μM, respectively.

## 3. Conclusions

Over the past five years, the field of phytochemistry has experienced a surge of progress, particularly in the study of grayanane diterpenes from the Ericaceae family. This period has been marked by the discovery of 193 novel diterpenes, each characterized by one of fifteen distinct carbon skeletons. This remarkable diversity not only underscores the richness of natural compounds but also highlights the ongoing potential for new and groundbreaking discoveries in this area. A significant focus of these studies has been on bioassay screenings, particularly evaluating in vivo pain activity using models like the acetic acid-induced writhing test. These tests have consistently demonstrated the potent analgesic properties of grayanane diterpenes. Additionally, certain compounds within this group have shown promising activity as inhibitors of PTP1B, suggesting potential therapeutic applications.

## 4. Future Perspectives

Looking to the future, the research into grayanane diterpenoids teems with exciting possibilities and opportunities. One critical area for future research is the detailed mechanistic study of these compounds, especially regarding their therapeutic applications [7]. Grayanane diterpenes are known for their potent toxicity, which is primarily attributed to their mechanism of action on the sodium channels in the nervous system, leading to a cascade of neurotoxic effects [7,37,38,39]. The limitations of using grayanane diterpenes stem from their narrow therapeutic index, the difficulty in controlling their dose-dependent toxic effects, and the potential for severe adverse reactions, including cardiac issues and central nervous system disturbances. Despite their potent bioactivity, which could be harnessed for therapeutic purposes, these limitations necessitate cautious handling and research to mitigate risks. Understanding the exact mode of action of grayanane diterpenes could revolutionize drug development and treatment strategies. This could lead to the creation of new drugs that harness the unique properties of these compounds, potentially offering more effective treatments for various conditions.

Another promising direction is the application of synthetic biology in the production of diterpenoids [40]. This approach could provide a sustainable and scalable alternative to traditional extraction methods from plants. This is particularly crucial for the large-scale production of these compounds, especially if they are to be used in therapeutic applications [41]. Synthetic biology might not only facilitate the production of these compounds but also enable the creation of novel diterpenoid derivatives with enhanced biological activities or reduced side effects.

Furthermore, exploring grayanane diterpenoids in combination therapies presents a significant opportunity for advancing medical treatments [42,43]. By combining these compounds with other drugs, there is potential to harness synergistic effects, which could lead to more effective treatments with fewer side effects. This approach aligns with the growing trend in pharmacology towards personalized medicine and treatment protocols that are more holistic and patient-specific. Moreover, exploring the broader range of biological activities of grayanane diterpenes is another avenue worth exploring. While much of the current research has focused on their analgesic and PTP1B inhibitory properties, these compounds may have other biological activities that are yet to be discovered. Investigating these potential activities could open up new therapeutic areas for these compounds.

In terms of technological advancements, the development of more sophisticated analytical techniques will play a crucial role in future research [44,45,46]. Technological advances such as mass spectrometry, NMR spectroscopy, and X-ray crystallography could lead to more detailed and accurate structural elucidation of these compounds. This, in turn, would enhance our understanding of their chemical properties and biological activities. The potential for international collaboration in this field also presents an exciting opportunity. By bringing together researchers from different countries and disciplines, the study of grayanane diterpenes can benefit from a wide range of expertise and resources. Such collaborations could lead to more rapid advancements in the field and sharing knowledge and techniques across borders.

In summary, the study of grayanane diterpenes stands at a pivotal point, with numerous avenues for future research and potential applications in pharmaceuticals and therapeutics. The continued exploration of these natural compounds is poised to significantly contribute to our understanding of natural product chemistry, medicinal chemistry, and pharmacology. As research progresses, grayanane diterpenes will likely play an increasingly important role in the development of new drugs and treatment strategies, highlighting the importance of natural products in modern medicine.

## Figures and Tables

**Table 1:** Compound Names, Plant Sources, Related References, and Year of Publication.

No.	Name	Plant Resource	Year	Ref.
**1**	Pierisformosoid A	*Pieris formosa*, roots	2018	[8]
**2**	Pierisformosoid B	*Pieris formosa*, roots	2018	[8]
**3**	Pierisformosoid C	*Pieris formosa*, roots	2018	[8]
**4**	Pierisformosoid D	*Pieris formosa*, roots	2018	[8]
**5**	Pierisformosoid E	*Pieris formosa*, roots	2018	[8]
**6**	Pierisformosoid F	*Pieris formosa*, roots	2018	[8]
**7**	Pierisformosoid G	*Pieris formosa*, roots	2018	[8]
**8**	Pierisformosoid H	*Pieris formosa*, roots	2018	[8]
**9**	Pierisformosoid I	*Pieris formosa*, roots	2018	[8]
**10**	Pierisformosoid J	*Pieris formosa*, roots	2018	[8]
**11**	Pierisformosoid K	*Pieris formosa*, roots	2018	[8]
**12**	Pierisformosoid L	*Pieris formosa*, roots	2018	[8]
**13**	3-epi-grayanoside B	*Rhododendron micranthum*, leaves	2018	[9]
**14**	Micranthanoside A	*Rhododendron micranthum*, leaves	2018	[9]
**15**	Micranthanoside B	*Rhododendron micranthum*, leaves	2018	[9]
**16**	Micranthanoside C	*Rhododendron micranthum*, leaves	2018	[9]
**17**	Micranthanoside D	*Rhododendron micranthum*, leaves	2018	[9]
**18**	Micranthanoside E	*Rhododendron micranthum*, leaves	2018	[9]
**19**	hydroxygrayanoside C	*Rhododendron micranthum*, leaves	2018	[9]
**20**	micranthanoside F	*Rhododendron micranthum*, leaves	2018	[9]
**21**	14β-acetyoxymicranthanoside	*Rhododendron micranthum*, leaves	2018	[9]
**22**	micranthanoside G	*Rhododendron micranthum*, leaves	2018	[9]
**23**	14-Oacetylmicranthanoside G	*Rhododendron micranthum*, leaves	2018	[9]
**24**	14β-hydroxypieroside A	*Rhododendron micranthum*, leaves	2018	[9]
**25**	micranthanoside H	*Rhododendron micranthum*, leaves	2018	[9]
**26**	Mollfoliagein D	*Rhododendron molle*, leaves	2018	[7]
**27**	6-O-Acetylrhodomollein XI	*Rhododendron molle*, leaves	2018	[7]
**28**	Mollfoliagein F	*Rhododendron molle*, leaves	2018	[7]
**29**	18-Hydroxygrayanotoxin XVIII	*Rhododendron molle*, leaves	2018	[7]
**30**	2-O-Methylrhodomolin I	*Rhododendron molle*, leaves	2018	[7]
**31**	2-O-Methylrhodomollein XII	*Rhododendron molle*, leaves	2018	[7]
**32**	2-O-Methylrhodojaponin VI	*Rhododendron molle*, leaves	2018	[7]
**33**	2-O-Methylrhodojaponin VII	*Rhododendron molle*, leaves	2018	[7]
**34**	Rhododecorumin VIII	*Rhododendron decorum*, leaves and twigs	2018	[22]
**35**	Rhododecorumin IX	*Rhododendron decorum*, leaves and twigs	2018	[22]
**36**	Rhododecorumin X	*Rhododendron decorum*, leaves and twigs	2018	[22]
**37**	Rhododecorumin XI	*Rhododendron decorum*, leaves and twigs	2018	[22]
**38**	Rhododecorumin XII	*Rhododendron decorum*, leaves and twigs	2018	[22]
**39**	Rhododeoside I	*Rhododendron decorum*, leaves and twigs	2018	[22]
**40**	Rhodoauriculatol I	*Rhododendron auriculatum*, leaves	2019	[21]
**41**	Rhodomicranoside F	*Rhododendron auriculatum*, leaves	2019	[14]
**42**	Rhodomicranoside G	*Rhododendron auriculatum*, leaves	2019	[14]
**43**	Rhodomicranoside H	*Rhododendron auriculatum*, leaves	2019	[14]
**44**	Rhodomicranoside I	*Rhododendron auriculatum*, leaves	2019	[14]
**45**	Auriculatol B	*Rhododendron auriculatum*, leaves	2019	[25]
**46**	3-epi-Grayanotoxin XVIII	*Rhododendron auriculatum*, leaves	2019	[25]
**47**	6-Deoxycraiobiotoxin I	*Rhododendron auriculatum*, leaves	2019	[25]
**48**	3-epi-Auriculatol B	*Rhododendron auriculatum*, leaves	2019	[25]
**49**	19-Hydroxy-3-epi-auriculatol B	*Rhododendron auriculatum*, leaves	2019	[25]
**50**	Auriculatol C	*Rhododendron auriculatum*, leaves	2019	[25]
**51**	Auriculatol D	*Rhododendron auriculatum*, leaves	2019	[25]
**52**	Auriculatol E	*Rhododendron auriculatum*, leaves	2019	[25]
**53**	Auriculatol F	*Rhododendron auriculatum*, leaves	2019	[25]
**54**	2α-Hydroxyauriculatol F	*Rhododendron auriculatum*, leaves	2019	[25]
**55**	1-epi-Pieristoxin S	*Rhododendron auriculatum*, leaves	2019	[25]
**56**	17-Hydroxygrayanotoxin XIX	*Pieris japonica*, leaves	2019	[26]
**57**	2-O-Methylrhodomollein XIX	*Pieris japonica*, leaves	2019	[26]
**58**	17-Hydroxy-3-epi-auriculatol B	*Pieris japonica*, leaves	2019	[26]
**59**	Pierisjaponol A	*Pieris japonica*, leaves	2019	[26]
**60**	Pierisjaponol B	*Pieris japonica*, leaves	2019	[26]
**61**	13α-Hydroxyrhodomollein XVII	*Pieris japonica*, leaves	2019	[26]
**62**	12β-Hydroxygrayanotoxin XVIII	*Pieris japonica*, leaves	2019	[26]
**63**	2α-Hydroxyasebotoxin II	*Pieris japonica*, leaves	2019	[26]
**64**	2α-O-Methylgrayanotoxin II	*Pieris japonica*, leaves	2019	[26]
**65**	Pierisjaponol C	*Pieris japonica*, leaves	2019	[26]
**66**	16-O-Methylgrayanotoxin XVIII	*Pieris japonica*, leaves	2019	[26]
**67**	Pierisjaponol D	*Pieris japonica*, leaves	2019	[26]
**68**	Rhodomollein XLIV	*Rhododendron molle*, flowers	2020	[20]
**69**	Rhodomollein XLV	*Rhododendron molle*, flowers	2020	[20]
**70**	Rhodomollein XLVI	*Rhododendron molle*, flowers	2020	[20]
**71**	Rhodomollein XLVII	*Rhododendron molle*, flowers	2020	[20]
**72**	Rhodomollein XLIX	*Rhododendron molle*, flowers	2020	[20]
**73**	Rhodomollein L	*Rhododendron molle*, flowers	2020	[20]
**74**	Dauricanol A	*Rhododendron dauricum*, flowers	2023	[16]
**75**	Dauricanol B	*Rhododendron dauricum*, flowers	2023	[16]
**76**	Dauricanol C	*Rhododendron dauricum*, flowers	2023	[16]
**77**	Daublossomin G	*Rhododendron dauricum*, flowers	2023	[27]
**78**	Daublossomin H	*Rhododendron dauricum*, flowers	2023	[27]
**79**	Daublossomin I	*Rhododendron dauricum*, flowers	2023	[27]
**80**	Daublossomin J	*Rhododendron dauricum*, flowers	2023	[27]
**81**	Daublossomin K	*Rhododendron dauricum*, flowers	2023	[27]
**82**	Daublossomin L	*Rhododendron dauricum*, flowers	2023	[27]
**83**	Daublossomin M	*Rhododendron dauricum*, flowers	2023	[27]
**84**	Craibiodenoside A	*Craibiodendron yunnanense*, leaves	2023	[28]
**85**	Craibiodenoside B	*Craibiodendron yunnanense*, leaves	2023	[28]
**86**	Craibiodenoside C	*Craibiodendron yunnanense*, leaves	2023	[28]
**87**	Molleblossomin G	*Rhododendron molle*, flowers	2024	[29]
**88**	Molleblossomin H	*Rhododendron molle*, flowers	2024	[29]
**89**	Molleblossomin I	*Rhododendron molle*, flowers	2024	[29]
**90**	Molleblossomin J	*Rhododendron molle*, flowers	2024	[29]
**91**	Molleblossomin K	*Rhododendron molle*, flowers	2024	[29]
**92**	Molleblossomin L	*Rhododendron molle*, flowers	2024	[29]
**93**	16-Acetylgrayanotoxin III	*Rhododendron micranthum*, roots	2020	[19]
**94**	3β, 6β, 16α-trihydroxy-14b-acetoxy-grayan- 1(5), 10(20)-diene	*Rhododendron micranthum*, roots	2020	[19]
**95**	14β-(2-Hydroxypropanoyloxy)rhodomollein XVII	*Craibiodendron yunnanense*, leaves	2023	[30]
**96**	2-O-Ethoxyrhodojaponin VI	*Craibiodendron yunnanense*, leaves	2023	[30]
**97**	Micranthanoside J	*Craibiodendron yunnanense*, leaves	2023	[30]
**98**	Mollfoliagein A	*Rhododendron molle*, leaves	2018	[7]
**99**	Mollfoliagein B	*Rhododendron molle*, leaves	2018	[7]
**100**	Mollfoliagein C	*Rhododendron molle*, leaves	2018	[7]
**101**	6-O-Acetylrhodomollein XXXI	*Rhododendron molle*, leaves	2018	[7]
**102**	Mollfoliagein E	*Rhododendron molle*, leaves	2018	[7]
**103**	Rhododecorumin VI	*Rhododendron decorum*, leaves and twigs	2018	[22]
**104**	Rhododecorumin VII	*Rhododendron decorum*, leaves and twigs	2018	[22]
**105**	Epoxypieristoxin A	*Pieris formosa*, roots	2019	[31]
**106**	Epoxypieristoxin B	*Pieris formosa*, roots	2019	[31]
**107**	Epoxypieristoxin C	*Pieris formosa*, roots	2019	[31]
**108**	Epoxypieristoxin D	*Pieris formosa*, roots	2019	[31]
**109**	Epoxypieristoxin E	*Pieris formosa*, roots	2019	[31]
**110**	Epoxypieristoxin F	*Pieris formosa*, roots	2019	[31]
**111**	Epoxypieristoxin G	*Pieris formosa*, roots	2019	[31]
**112**	Epoxypieristoxin H	*Pieris formosa*, roots	2019	[31]
**113**	14-Deoxyrhodomollein XXXVII	*Pieris japonica*, leaves	2019	[26]
**114**	Rhodomollein XLVIII	*Rhododendron molle*, flowers	2020	[20]
**115**	Micranthanol A	*Rhododendron micranthum*, leaves	2021	[17]
**116**	Micranthanol B	*Rhododendron micranthum*, leaves	2021	[17]
**117**	Daublossomin A	*Rhododendron dauricum*, flowers	2023	[27]
**118**	Daublossomin B	*Rhododendron dauricum*, flowers	2023	[27]
**119**	Daublossomin C	*Rhododendron dauricum*, flowers	2023	[27]
**120**	Daublossomin D	*Rhododendron dauricum*, flowers	2023	[27]
**121**	Daublossomin E	*Rhododendron dauricum*, flowers	2023	[27]
**122**	Daublossomin F	*Rhododendron dauricum*, flowers	2023	[27]
**123**	Craibiodenoside D	*Craibiodendron yunnanense*, leaves	2023	[28]
**124**	Craibiodenoside E	*Craibiodendron yunnanense*, leaves	2023	[28]
**125**	Craibiodenoside F	*Craibiodendron yunnanense*, leaves	2023	[28]
**126**	Molleblossomin A	*Rhododendron molle*, flowers	2024	[29]
**127**	Molleblossomin B	*Rhododendron molle*, flowers	2024	[29]
**128**	Molleblossomin C	*Rhododendron molle*, flowers	2024	[29]
**129**	Molleblossomin D	*Rhododendron molle*, flowers	2024	[29]
**130**	Molleblossomin E	*Rhododendron molle*, flowers	2024	[29]
**131**	Molleblossomin F	*Rhododendron molle*, flowers	2024	[29]
**132**	Auriculatol A	*Rhododendron auriculatum*, leaves	2019	[25]
**133**	9β-Hydroxy-1,5-seco-grayanotoxin	*Rhododendron micranthum*, leaves	2021	[17]
**134**	Dauricanol D	*Rhododendron dauricum*, flowers	2023	[16]
**135**	Dauricanol E	*Rhododendron dauricum*, flowers	2023	[16]
**136**	Pierisjaponin A	*Pieris japonica*, leaves	2020	[18]
**137**	Pierisjaponin B	*Pieris japonica*, leaves	2020	[18]
**138**	Rhodoauriculatol A	*Rhododendron auriculatum*, leaves	2019	[21]
**139**	Rhodoauriculatol B	*Rhododendron auriculatum*, leaves	2019	[21]
**140**	Rhodoauriculatol C	*Rhododendron auriculatum*, leaves	2019	[21]
**141**	Rhodoauriculatol D	*Rhododendron auriculatum*, leaves	2019	[21]
**142**	Pierisjaponin J	*Pieris japonica*, leaves	2020	[18]
**143**	Birhodomollein D	*Rhododendron pumilum*, fruits	2018	[32]
**144**	Birhodomollein E	*Rhododendron pumilum*, fruits	2018	[32]
**145**	Bimollfoliagein A	*Rhododendron molle*, leaves	2018	[7]
**146**	Rhodomollein XLIII	*Rhododendron molle*, flowers	2020	[20]
**147**	Bismollether A	*Rhododendron molle*, flowers	2022	[24]
**148**	Bismollether B	*Rhododendron molle*, flowers	2022	[24]
**149**	Bismollether C	*Rhododendron molle*, flowers	2022	[24]
**150**	Rhododecorumin I	*Rhododendron decorum*, leaves and twigs	2018	[22]
**151**	Rhododecorumin II	*Rhododendron decorum*, leaves and twigs	2018	[22]
**152**	Rhododecorumin III	*Rhododendron decorum*, leaves and twigs	2018	[22]
**153**	Rhodoauriculatol G	*Rhododendron auriculatum*, leaves	2019	[21]
**154**	Rhodoauriculatol H	*Rhododendron auriculatum*, leaves	2019	[21]
**155**	Rhodomicranoside A	*Rhododendron auriculatum*, leaves	2019	[14]
**156**	Rhodomicranoside B	*Rhododendron auriculatum*, leaves	2019	[14]
**157**	Rhodomicranoside C	*Rhododendron auriculatum*, leaves	2019	[14]
**158**	Rhodomollein LII	*Rhododendron molle*, flowers	2020	[20]
**159**	Rhodomollein LIII	*Rhododendron molle*, flowers	2020	[20]
**160**	3β,7α,14β-trihydroxy-leucoth-10(20),15-dien-5-one	*Pieris formosa*, roots	2020	[15]
**161**	10α,16α-dihydroxy-leucoth-5-one	*Pieris formosa*, roots	2020	[15]
**162**	Pierisjaponin F	*Pieris japonica*, leaves	2020	[18]
**163**	Pierisjaponin G	*Pieris japonica*, leaves	2020	[28]
**164**	Rhodoauriculatol F	*Rhododendron auriculatum*, leaves	2019	[21]
**165**	Pierisentkauran B	*Pieris formosa*, roots	2020	[15]
**166**	Pierisentkauran C	*Pieris formosa*, roots	2020	[15]
**167**	Pierisentkauran D	*Pieris formosa*, roots	2020	[15]
**168**	Pierisentkauran E	*Pieris formosa*, roots	2020	[15]
**169**	Rhodomicranoside D	*Rhododendron micranthum*, leaves	2019	[14]
**170**	Rhodomicranoside E	*Rhododendron micranthum*, leaves	2019	[14]
**171**	Pierisentkauran F	*Pieris formosa*, roots	2020	[15]
**172**	Pierisjaponin H	*Pieris japonica*, leaves	2020	[18]
**173**	Pierisjaponin I	*Pieris japonica*, leaves	2020	[18]
**174**	8α-O-Acetylrhodomollein XXIII	*Rhododendron micranthum*, leaves	2021	[17]
**175**	Rhodokalmanol A	*Rhododendron dauricum*, leaves	2022	[33]
**176**	Rhodokalmanol B	*Rhododendron dauricum*, leaves	2022	[33]
**177**	Rhodokalmanol C	*Rhododendron dauricum*, leaves	2022	[33]
**178**	Rhodokalmanol D	*Rhododendron dauricum*, leaves	2022	[33]
**179**	16α-acetoxy rhodomollein XXIII	*Rhododendron micranthum*, roots	2020	[19]
**180**	Rhodomollein LI	*Rhododendron molle*, flowers	2020	[20]
**181**	Rhodoauriculatol E	*Rhododendron auriculatum*, leaves	2019	[21]
**182**	Mollebenzylanol A	*Rhododendron molle*, leaves	2018	[23]
**183**	Mollebenzylanol B	*Rhododendron molle*, leaves	2018	[23]
**184**	Rhododecorumin IV	*Rhododendron decorum*, leaves and twigs	2018	[22]
**185**	Rhododecorumin V	*Rhododendron decorum*, leaves and twigs	2018	[22]
**186**	Micranthanone B	*Rhododendron micranthum*, leaves	2021	[17]
**187**	Micranthanone C	*Rhododendron micranthum*, leaves	2021	[17]
**188**	14-epi-Mollanol A	*Rhododendron micranthum*, leaves	2021	[17]
**189**	Mollanol B	*Rhododendron micranthum*, leaves	2021	[17]
**190**	Mollanol C	*Rhododendron micranthum*, leaves	2021	[17]
**191**	Pierisjaponin E	*Pieris japonica*, leaves	2020	[18]
**192**	Rhomollone A	*Rhododendron molle*, flowers	2020	[20]
**193**	rhodauricanol A	*Rhododendron dauricum*, flowers	2023	[16]

**Table 2:** Compound Names and Their Reported Activities.

No	In Vivo		In Vitro	
	Test Mode	Activity/Dose	Test Model	Activity/Dose
**1**	Acetic acid-induced pain mouse model *Plutella xylostella*	Analgesic, 5 mg/kg Antifeedant, 0.5 mg/mL	Nav1.7 channel KCNQ2 channel	ND, 10 μM ND, 10 μM
**2**	Acetic acid-induced pain mouse model	Analgesic, 1 mg/kg	Nav1.7 channel KCNQ2 channel	ND, 10 μM ND, 10 μM
**3**	-	-	Nav1.7 channel KCNQ2 channel	ND, 10 μM ND, 10 μM
**4**	Acetic acid-induced pain mouse model *Plutella xylostella*	Analgesic, 0.1 mg/kg Antifeedant, 0.5 mg/mL	Nav1.7 channel KCNQ2 channel	ND, 10 μM 38.3% inhibitory, 10 μM
**5**	Acetic acid-induced pain mouse model	Analgesic, 5 mg/kg	Nav1.7 channel KCNQ2 channel	ND, 10 μM ND, 10 μM
**6**	-	-	Nav1.7 channel KCNQ2 channel	ND, 10 μM ND, 10 μM
**7**	Acetic acid-induced pain mouse model	Analgesic, 0.1 mg/kg	Nav1.7 channel KCNQ2 channel	ND, 10 μM ND, 10 μM
**8**	Acetic acid-induced pain mouse model	Analgesic, 5 mg/kg	Nav1.7 channel KCNQ2 channel	ND, 10 μM ND, 10 μM
**9**	Acetic acid-induced pain mouse model Plutella xylostella	ND Antifeedant, 0.5 mg/mL	Nav1.7 channel KCNQ2 channel	ND, 10 μM ND, 10 μM
**10**	Acetic acid-induced pain mouse model	ND	Nav1.7 channel KCNQ2 channel	ND, 10 μM ND, 10 μM
**11**	Acetic acid-induced pain mouse model	ND	Nav1.7 channel KCNQ2 channel	ND, 10 μM ND, 10 μM
**12**	Acetic acid-induced pain mouse model	ND	Nav1.7 channel KCNQ2 channel	ND, 10 μM ND, 10 μM
**13**	Acetic acid-induced pain mouse model	Analgesic, 5.0 mg/kg	Anti-inflammatory Cytotoxicity PTP1B	ND, 40 μM ND, 40 μM ND, 40 μM
**14**	Acetic acid-induced pain mouse model	Analgesic, 0.2 mg/kg	Anti-inflammatory Cytotoxicity PTP1B	ND, 40 μM ND, 40 μM ND, 40 μM
**15**	Acetic acid-induced pain mouse model	Analgesic, 1.0 mg/kg	Anti-inflammatory Cytotoxicity PTP1B	ND, 40 μM ND, 40 μM ND, 40 μM
**16**	Acetic acid-induced pain mouse model	Analgesic, 5.0 mg/kg	Anti-inflammatory Cytotoxicity PTP1B	ND, 40 μM ND, 40 μM ND, 40 μM
**17**	Acetic acid-induced pain mouse model	Analgesic, 5.0 mg/kg	Anti-inflammatory Cytotoxicity PTP1B	ND, 40 μM ND, 40 μM ND, 40 μM
**18**	Acetic acid-induced pain mouse model	Analgesic, 5.0 mg/kg	Anti-inflammatory Cytotoxicity PTP1B	ND, 40 μM ND, 40 μM ND, 40 μM
**19**	Acetic acid-induced pain mouse model	Analgesic, 1.0 mg/kg	Anti-inflammatory Cytotoxicity PTP1B	ND, 40 μM ND, 40 μM ND, 40 μM
**20**	Acetic acid-induced pain mouse model	Analgesic, 5.0 mg/kg	Anti-inflammatory Cytotoxicity PTP1B	ND, 40 μM ND, 40 μM ND, 40 μM
**21**	Acetic acid-induced pain mouse model	Analgesic, 5.0 mg/kg	Anti-inflammatory Cytotoxicity PTP1B	ND, 40 μM ND, 40 μM ND, 40 μM
**22**	Acetic acid-induced pain mouse model	Analgesic, 5.0 mg/kg	Anti-inflammatory Cytotoxicity PTP1B	ND, 40 μM ND, 40 μM ND, 40 μM
**23**	Acetic acid-induced pain mouse model	Analgesic, 5.0 mg/kg	Anti-inflammatory Cytotoxicity PTP1B	ND, 40 μM ND, 40 μM ND, 40 μM
**24**	Acetic acid-induced pain mouse model	Analgesic, 5.0 mg/kg	Anti-inflammatory Cytotoxicity PTP1B	ND, 40 μM ND, 40 μM ND, 40 μM
**25**	Acetic acid-induced pain mouse model	Analgesic, 5.0 mg/kg	Anti-inflammatory Cytotoxicity PTP1B	ND, 40 μM ND, 40 μM ND, 40 μM
**26**	-		Anti-inflammatory	ND, 40 μM
**27**	-		Anti-inflammatory	ND, 40 μM
**28**	-		Anti-inflammatory	ND, 40 μM
**29**	-		Anti-inflammatory	ND, 40 μM
**30**	-		Anti-inflammatory	ND, 40 μM
**31**	-		Anti-inflammatory	ND, 40 μM
**32**	-		Anti-inflammatory	ND, 40 μM
**33**	-		Anti-inflammatory	ND, 40 μM
**34**	Acetic acid-induced pain mouse model	Analgesic, 10.0 mg/kg	-	
**35**	-		-	
**36**	Acetic acid-induced pain mouse model	Analgesic, 10.0 mg/kg	-	
**37**	Acetic acid-induced pain mouse model	Analgesic, 10.0 mg/kg	-	
**38**	Acetic acid-induced pain mouse model	Analgesic, 0.8 mg/kg	-	
**39**	Acetic acid-induced pain mouse model	Analgesic, 10.0 mg/kg	-	
**40**	Acetic acid-induced pain mouse model	Analgesic, 1.0 mg/kg	-	
**41**	Acetic acid-induced pain mouse model	Analgesic, 5.0 mg/kg	-	
**42**	Acetic acid-induced pain mouse model	Analgesic, 5.0 mg/kg	-	
**43**	Acetic acid-induced pain mouse model	Analgesic, 1.0 mg/kg	-	
**44**	Acetic acid-induced pain mouse model	Analgesic, 1.0 mg/kg	-	
**45**	Acetic acid-induced pain mouse model	Analgesic, 5.0 mg/kg	-	
**46**	Acetic acid-induced pain mouse model	Analgesic, 5.0 mg/kg	-	
**47**	Acetic acid-induced pain mouse model	Analgesic, 5.0 mg/kg	-	
**48**	Acetic acid-induced pain mouse model	Analgesic, 5.0 mg/kg	-	
**49**	Acetic acid-induced pain mouse model	Analgesic, 5.0 mg/kg	-	
**50**	Acetic acid-induced pain mouse model	Analgesic, 5.0 mg/kg	-	
**51**	Acetic acid-induced pain mouse model	Analgesic, 5.0 mg/kg	-	
**52**	Acetic acid-induced pain mouse model	Analgesic, 1.0 mg/kg	-	
**53**	Acetic acid-induced pain mouse model	Analgesic, 5.0 mg/kg	-	
**54**	Acetic acid-induced pain mouse model	Analgesic, 5.0 mg/kg	-	
**55**	Acetic acid-induced pain mouse model	Analgesic, 5.0 mg/kg	-	
**56**	Acetic acid-induced pain mouse model	Analgesic, 0.04 mg/kg	-	
**57**	Acetic acid-induced pain mouse model	Analgesic, 5.0 mg/kg	-	
**58**	Acetic acid-induced pain mouse model	Analgesic, 5.0 mg/kg	-	
**59**	Acetic acid-induced pain mouse model	Analgesic, 0.2 mg/kg	-	
**60**	Acetic acid-induced pain mouse model	Analgesic, 5.0 mg/kg	-	
**61**	Acetic acid-induced pain mouse model	Analgesic, 5.0 mg/kg	-	
**62**	Acetic acid-induced pain mouse model	Analgesic, 5.0 mg/kg	-	
**63**	Acetic acid-induced pain mouse model	Analgesic, 5.0 mg/kg	-	
**64**	Acetic acid-induced pain mouse model	Analgesic, 5.0 mg/kg	-	
**65**	Acetic acid-induced pain mouse model	Analgesic, 5.0 mg/kg	-	
**66**	Acetic acid-induced pain mouse model	Analgesic, 5.0 mg/kg	-	
**67**	Acetic acid-induced pain mouse model	Analgesic, 5.0 mg/kg	-	
**68**	Acetic acid-induced pain mouse model	Analgesic, 20.0 mg/kg	-	
**69**	Acetic acid-induced pain mouse model	Analgesic, 20.0 mg/kg	-	
**70**	-		-	
**71**	Acetic acid-induced pain mouse model	Analgesic, 2.0 mg/kg	-	
**72**	-		-	
**73**	-		-	
**74**	Acetic acid-induced pain mouse model	Analgesic, 5.0 mg/kg	-	
**75**	Acetic acid-induced pain mouse model	Analgesic, 0.04 mg/kg	-	
**76**	Acetic acid-induced pain mouse model	Analgesic, 0.04 mg/kg	-	
**77**	Acetic acid-induced pain mouse model	Analgesic, 5.0 mg/kg	-	
**78**	Acetic acid-induced pain mouse model	Analgesic, 5.0 mg/kg	-	
**79**	Acetic acid-induced pain mouse model	ND	-	
**80**	Acetic acid-induced pain mouse model	Analgesic, 5.0 mg/kg	-	
**81**	Acetic acid-induced pain mouse model	Analgesic, 5.0 mg/kg	-	
**82**	-		-	
**83**	Acetic acid-induced pain mouse model	Analgesic, 5.0 mg/kg	-	
**84**	-		Anti-inflammatory	ND, 10 μg/mL
**85**	-		Anti-inflammatory	10 μg/mL
**86**	-		Anti-inflammatory	10 μg/mL
**87**	Acetic acid-induced pain mouse model	Analgesic, 5.0 mg/kg	-	
**88**	Acetic acid-induced pain mouse model	Analgesic, 5.0 mg/kg	-	
**89**	Acetic acid-induced pain mouse model	Analgesic, 5.0 mg/kg	-	
**90**	Acetic acid-induced pain mouse model	Analgesic, 5.0 mg/kg	-	
**91**	Acetic acid-induced pain mouse model	Analgesic, 5.0 mg/kg	-	
**92**	Acetic acid-induced pain mouse model	Analgesic, 5.0 mg/kg	-	
**93**	Acetic acid-induced pain mouse model	Analgesic, 1.0 mg/kg	-	
**94**	Acetic acid-induced pain mouse model	Analgesic, 0.8 mg/kg	-	
**95**	-		-	
**96**	-		-	
**97**	-		-	
**98**	-		Anti-inflammatory	ND, 40 μM
**99**	-		Anti-inflammatory	ND, 40 μM
**100**	-		Anti-inflammatory	IC_50_ 35.4 ± 3.9 μM
**101**	-		Anti-inflammatory	ND, 40 μM
**102**	-		Anti-inflammatory	ND, 40 μM
**103**	Acetic acid-induced pain mouse model	Analgesic, 10.0 mg/kg	-	
**104**	-		-	
**105**	Acetic acid-induced pain mouse model	Analgesic, 5.0 mg/kg	-	
**106**	Acetic acid-induced pain mouse model	Analgesic, 5.0 mg/kg	-	
**107**	Acetic acid-induced pain mouse model	Analgesic, 5.0 mg/kg	-	
**108**	-		-	
**109**	Acetic acid-induced pain mouse model	Analgesic, 5.0 mg/kg	-	
**110**	Acetic acid-induced pain mouse model	Analgesic, 5.0 mg/kg	-	
**111**	Acetic acid-induced pain mouse model	Analgesic, 5.0 mg/kg	-	
**112**	Acetic acid-induced pain mouse model	Analgesic, 5.0 mg/kg	-	
**113**	Acetic acid-induced pain mouse model	Analgesic, 5.0 mg/kg	-	
**114**	Acetic acid-induced pain mouse model	Analgesic, 20.0 mg/kg	-	
**115**	Acetic acid-induced pain mouse model	Analgesic, 5.0 mg/kg	-	
**116**	Acetic acid-induced pain mouse model	Analgesic, 5.0 mg/kg	-	
**117**	Acetic acid-induced pain mouse model	Analgesic, 0.2 mg/kg	-	
**118**	Acetic acid-induced pain mouse model	Analgesic, 5.0 mg/kg	-	
**119**	Acetic acid-induced pain mouse model	Analgesic, 5.0 mg/kg	-	
**120**	Acetic acid-induced pain mouse model	Analgesic, 5.0 mg/kg	-	
**121**	Acetic acid-induced pain mouse model	Analgesic, 5.0 mg/kg	-	
**122**	Acetic acid-induced pain mouse model	Analgesic, 0.2 mg/kg	-	
**123**	-		Anti-inflammatory	ND, 10 μg/mL
**124**	-		Anti-inflammatory	ND, 10 μg/mL
**125**	-		Anti-inflammatory	10 μg/mL
**126**	Acetic acid-induced pain mouse model	Analgesic, 5.0 mg/kg	-	
**127**	Acetic acid-induced pain mouse model	Analgesic, 5.0 mg/kg	-	
**128**	Acetic acid-induced pain mouse model	Analgesic, 5.0 mg/kg	-	
**129**	Acetic acid-induced pain mouse model	Analgesic, 5.0 mg/kg	-	
**130**	Acetic acid-induced pain mouse model	Analgesic, 5.0 mg/kg	-	
**131**	Acetic acid-induced pain mouse model	Analgesic, 0.2 mg/kg	-	
**132**	Acetic acid-induced pain mouse model	Analgesic, 5.0 mg/kg	-	
**133**	Acetic acid-induced pain mouse model	Analgesic, 5.0 mg/kg	-	
**134**	Acetic acid-induced pain mouse model	Analgesic, 1.0 mg/kg	-	
**135**	Acetic acid-induced pain mouse model	Analgesic, 1.0 mg/kg	-	
**136**	Acetic acid-induced pain mouse model	Analgesic, 1.0 mg/kg	-	
**137**	Acetic acid-induced pain mouse model	Analgesic, 0.04 mg/kg	-	
**138**	Acetic acid-induced pain mouse model	Analgesic, 5.0 mg/kg	-	
**139**	Acetic acid-induced pain mouse model	Analgesic, 5.0 mg/kg	-	
**140**	Acetic acid-induced pain mouse model	Analgesic, 5.0 mg/kg	-	
**141**	Acetic acid-induced pain mouse model	Analgesic, 5.0 mg/kg	-	
**142**	Acetic acid-induced pain mouse model	Analgesic, 5.0 mg/kg	-	
**143**	-		-	
**144**	-		-	
**145**	-		Anti-inflammatory	ND, 40 μM
**146**	-		-	
**147**	Acetic acid-induced pain mouse model	Analgesic, 5.0 mg/kg	-	
**148**	Acetic acid-induced pain mouse model Capsaicin-induced pain mouse model AITC-induced pain mouse model	Analgesic, 0.2 mg/kg Analgesic, 5.0 mg/kg Analgesic, 5.0 mg/kg	-	
**149**	Acetic acid-induced pain mouse model	Analgesic, 5.0 mg/kg		
**150**	Acetic acid-induced pain mouse model	Analgesic, 10.0 mg/kg	-	
**151**	-		-	
**152**	Acetic acid-induced pain mouse model	Analgesic, 10.0 mg/kg	-	
**153**	Acetic acid-induced pain mouse model	Analgesic, 5.0 mg/kg	-	
**154**	Acetic acid-induced pain mouse model	Analgesic, 5.0 mg/kg	-	
**155**	Acetic acid-induced pain mouse model	Analgesic, 1.0 mg/kg	-	
**156**	Acetic acid-induced pain mouse model	Analgesic, 1.0 mg/kg	-	
**157**	Acetic acid-induced pain mouse model	Analgesic, 1.0 mg/kg	-	
**158**	-		-	
**159**	Acetic acid-induced pain mouse model	Analgesic, 5.0 mg/kg	-	
**160**	Acetic acid-induced pain mouse model	Analgesic, 5.0 mg/kg	-	
**161**	Acetic acid-induced pain mouse model	Analgesic, 5 mg/kg Antifeedant, 0.5 mg/mL	-	
**162**	Acetic acid-induced pain mouse model	Analgesic, 1.0 mg/kg	-	
**163**	Acetic acid-induced pain mouse model	Analgesic, 5.0 mg/kg	-	
**164**	Acetic acid-induced pain mouse model	Analgesic, 5.0 mg/kg	-	
**165**	-		-	
**166**	Acetic acid-induced pain mouse model	Analgesic, 5.0 mg/kg	-	
**167**	*Plutella xylostella*	Antifeedant, 0.5 mg/mL	-	
**168**	-		-	
**169**	Acetic acid-induced pain mouse model	Analgesic, 1.0 mg/kg	-	
**170**	Acetic acid-induced pain mouse model	Analgesic, 1.0 mg/kg	-	
**171**	Acetic acid-induced pain mouse model	Analgesic, 5.0 mg/kg	-	
**172**	Acetic acid-induced pain mouse model	Analgesic, 5.0 mg/kg	-	
**173**	Acetic acid-induced pain mouse model	Analgesic, 1.0 mg/kg	-	
**174**	Acetic acid-induced pain mouse model	Analgesic, 5.0 mg/kg	-	
**175**	Acetic acid-induced pain mouse model	Analgesic, 1.0 mg/kg	-	
**176**	Acetic acid-induced pain mouse model	Analgesic, 5.0 mg/kg	-	
**177**	Acetic acid-induced pain mouse model	Analgesic, 0.04 mg/kg	-	
**178**	Acetic acid-induced pain mouse model	Analgesic, 0.2 mg/kg	-	
**179**	Acetic acid-induced pain mouse model	Analgesic, 1.0 mg/kg	-	
**180**	-		-	
**181**	Acetic acid-induced pain mouse model	Analgesic, 5.0 mg/kg	-	
**182**	-		PTP1B	IC_50_ 22.99 ± 0.43 μM
**183**	-		PTP1B	IC_50_ 32.24 ± 0.74 μM
**184**	Acetic acid-induced pain mouse model	Analgesic, 10.0 mg/kg	-	
**185**	-		-	
**186**	Acetic acid-induced pain mouse model	Analgesic, 1.0 mg/kg	-	
**187**	Acetic acid-induced pain mouse model	Analgesic, 1.0 mg/kg	-	
**188**	Acetic acid-induced pain mouse model	Analgesic, 5.0 mg/kg	-	
**189**	Acetic acid-induced pain mouse model	Analgesic, 5.0 mg/kg	-	
**190**	Acetic acid-induced pain mouse model	Analgesic, 5.0 mg/kg	-	
**191**	Acetic acid-induced pain mouse model	Analgesic, 5.0 mg/kg	-	
**192**	-		-	
**193**	Acetic acid-induced pain mouse model	Analgesic, 0.2 mg/kg	-	

ND: Inactive at the tested concentration; -: Did not test.

## Supplementary Materials

The following supporting information can be downloaded at: https://www.mdpi.com/article/10.3390/molecules29071649/s1, Table S1. Compound names, plant resources, related references, and published year; Table S2. Compound names and reported activities.

## Abbreviations

AITC	Allyl isothiocyanate
LPS	lipopolysaccharide
NMR	Nuclear magnetic resonance
ECD	Electronic circular dichroism
PTP1B	Protein tyrosine phosphatase 1B
*C. yunnanense*	*Craibiodendron yunnanense*
*P. formosa*	*Pieris formosa*
*R. micranthum*	*Rhododendron micranthum*
*R. molle*	*Rhododendron molle*
*R. decorum*	*Rhododendron decorum*
*R. auriculatum*	*Rhododendron auriculatum*
*P. japonica*	*Pieris japonica*
*R. dauricum*	*Rhododendron dauricum*
*R. pumilum*	*Rhododendron pumilum*
