# Supplementary material for: Recent Advances in Grayanane Diterpenes: Isolation, Structural Diversity, and Bioactivities from Ericaceae Family (2018–2024)
Source: Molecules. 2024 Apr 6;29(7):1649. doi: 10.3390/molecules29071649 (PMC11013853; doi:10.3390/molecules29071649)
Supplement: Supplementary file 1 [file molecules-29-01649-s001.zip › molecules-2915220-supplementary.pdf]

Table S1. Compound names, plant resources, related references, and published year.

No.	Name	Plant resource	Year	ref
**1**	Pierisformosoid A	*Pieris formosa*, roots	2018	8
**2**	Pierisformosoid B	*Pieris formosa*, roots	2018	8
**3**	Pierisformosoid C	*Pieris formosa*, roots	2018	8
**4**	Pierisformosoid D	*Pieris formosa*, roots	2018	8
**5**	Pierisformosoid E	*Pieris formosa*, roots	2018	8
**6**	Pierisformosoid F	*Pieris formosa*, roots	2018	8
**7**	Pierisformosoid G	*Pieris formosa*, roots	2018	8
**8**	Pierisformosoid H	*Pieris formosa*, roots	2018	8
**9**	Pierisformosoid I	*Pieris formosa*, roots	2018	8
**10**	Pierisformosoid J	*Pieris formosa*, roots	2018	8
**11**	Pierisformosoid K	*Pieris formosa*, roots	2018	8
**12**	Pierisformosoid L	*Pieris formosa*, roots	2018	8
**13**	3-epi-grayanoside B	*Rhododendron micranthum*, leaves	2018	9
**14**	Micranthanoside A	*Rhododendron micranthum*, leaves	2018	9
**15**	Micranthanoside B	*Rhododendron micranthum*, leaves	2018	9
**16**	Micranthanoside C	*Rhododendron micranthum*, leaves	2018	9
**17**	Micranthanoside D	*Rhododendron micranthum*, leaves	2018	9
**18**	Micranthanoside E	*Rhododendron micranthum*, leaves	2018	9
**19**	hydroxygrayanoside C	*Rhododendron micranthum*, leaves	2018	9
**20**	micranthanoside F	*Rhododendron micranthum*, leaves	2018	9
**21**	14β-acetyoxymicranthanoside	*Rhododendron micranthum*, leaves	2018	9
**22**	micranthanoside G	*Rhododendron micranthum*, leaves	2018	9
**23**	14-Oacetylmicranthanoside G	*Rhododendron micranthum*, leaves	2018	9
**24**	14β-hydroxypieroside A	*Rhododendron micranthum*, leaves	2018	9
**25**	micranthanoside H	*Rhododendron micranthum*, leaves	2018	9
**26**	Mollfoliagein D	*Rhododendron molle*, leaves	2018	7
**27**	6-O-Acetylrhodomollein XI	*Rhododendron molle*, leaves	2018	7
**28**	Mollfoliagein F	*Rhododendron molle*, leaves	2018	7
**29**	18-Hydroxygrayanotoxin XVIII	*Rhododendron molle*, leaves	2018	7
**30**	2-O-Methylrhodomolin I	*Rhododendron molle*, leaves	2018	7
**31**	2-O-Methylrhodomollein XII	*Rhododendron molle*, leaves	2018	7
**32**	2-O-Methylrhodojaponin VI	*Rhododendron molle*, leaves	2018	7
**33**	2-O-Methylrhodojaponin VII	*Rhododendron molle*, leaves	2018	7
**34**	Rhododecorumin VIII	*Rhododendron decorum*, leaves and twigs	2018	22
**35**	Rhododecorumin IX	*Rhododendron decorum*, leaves and twigs	2018	22
**36**	Rhododecorumin X	*Rhododendron decorum*, leaves and twigs	2018	22
**37**	Rhododecorumin XI	*Rhododendron decorum*, leaves and twigs	2018	22
**38**	Rhododecorumin XII	*Rhododendron decorum*, leaves and twigs	2018	22
**39**	Rhododeoside I	*Rhododendron decorum*, leaves and twigs	2018	22
**40**	Rhodoauriculatol I	*Rhododendron auriculatum*, leaves	2019	21
**41**	Rhodomicranoside F	*Rhododendron auriculatum*, leaves	2019	14
**42**	Rhodomicranoside G	*Rhododendron auriculatum*, leaves	2019	14
**43**	Rhodomicranoside H	*Rhododendron auriculatum*, leaves	2019	14
**44**	Rhodomicranoside I	*Rhododendron auriculatum*, leaves	2019	14
**45**	Auriculatol B	*Rhododendron auriculatum*, leaves	2019	25
**46**	3-epi-Grayanotoxin XVIII	*Rhododendron auriculatum*, leaves	2019	25
**47**	6-Deoxycraiobiotoxin I	*Rhododendron auriculatum*, leaves	2019	25
**48**	3-epi-Auriculatol B	*Rhododendron auriculatum*, leaves	2019	25
**49**	19-Hydroxy-3-epi-auriculatol B	*Rhododendron auriculatum*, leaves	2019	25
**50**	Auriculatol C	*Rhododendron auriculatum*, leaves	2019	25
**51**	Auriculatol D	*Rhododendron auriculatum*, leaves	2019	25
**52**	Auriculatol E	*Rhododendron auriculatum*, leaves	2019	25
**53**	Auriculatol F	*Rhododendron auriculatum*, leaves	2019	25

**54**	2α-Hydroxyauriculatol F	*Rhododendron auriculatum*, leaves	2019	25
**55**	1-epi-Pieristoxin S	*Rhododendron auriculatum*, leaves	2019	25
**56**	17-Hydroxygrayanotoxin XIX	*Pieris japonica*, leaves	2019	26
**57**	2-O-Methylrhodomollein XIX	*Pieris japonica*, leaves	2019	26
**58**	17-Hydroxy-3-epi-auriculatol B	*Pieris japonica*, leaves	2019	26
**59**	Pierisjaponol A	*Pieris japonica*, leaves	2019	26
**60**	Pierisjaponol B	*Pieris japonica*, leaves	2019	26
**61**	13α-Hydroxyrhodomollein XVII	*Pieris japonica*, leaves	2019	26
**62**	12β-Hydroxygrayanotoxin XVIII	*Pieris japonica*, leaves	2019	26
**63**	2α-Hydroxyasebotoxin II	*Pieris japonica*, leaves	2019	26
**64**	2α-O-Methylgrayanotoxin II	*Pieris japonica*, leaves	2019	26
**65**	Pierisjaponol C	*Pieris japonica*, leaves	2019	26
**66**	16-O-Methylgrayanotoxin XVIII	*Pieris japonica*, leaves	2019	26
**67**	Pierisjaponol D	*Pieris japonica*, leaves	2019	26
**68**	Rhodomollein XLIV	*Rhododendron molle*, flowers	2020	20
**69**	Rhodomollein XLV	*Rhododendron molle*, flowers	2020	20
**70**	Rhodomollein XLVI	*Rhododendron molle*, flowers	2020	20
**71**	Rhodomollein XLVII	*Rhododendron molle*, flowers	2020	20
**72**	Rhodomollein XLIX	*Rhododendron molle*, flowers	2020	20
**73**	Rhodomollein L	*Rhododendron molle*, flowers	2020	20
**74**	Dauricanol A	*Rhododendron dauricum*, flowers	2023	16
**75**	Dauricanol B	*Rhododendron dauricum*, flowers	2023	16
**76**	Dauricanol C	*Rhododendron dauricum*, flowers	2023	16
**77**	Daublossomin G	*Rhododendron dauricum*, flowers	2023	27
**78**	Daublossomin H	*Rhododendron dauricum*, flowers	2023	27
**79**	Daublossomin I	*Rhododendron dauricum*, flowers	2023	27
**80**	Daublossomin J	*Rhododendron dauricum*, flowers	2023	27
**81**	Daublossomin K	*Rhododendron dauricum*, flowers	2023	27
**82**	Daublossomin L	*Rhododendron dauricum*, flowers	2023	27
**83**	Daublossomin M	*Rhododendron dauricum*, flowers	2023	27
**84**	Craibiodenoside A	*Craibiodendron yunnanense*, leaves	2023	28
**85**	Craibiodenoside B	*Craibiodendron yunnanense*, leaves	2023	28
**86**	Craibiodenoside C	*Craibiodendron yunnanense*, leaves	2023	28
**87**	Molleblossomin G	*Rhododendron molle*, flowers	2024	29
**88**	Molleblossomin H	*Rhododendron molle*, flowers	2024	29
**89**	Molleblossomin I	*Rhododendron molle*, flowers	2024	29
**90**	Molleblossomin J	*Rhododendron molle*, flowers	2024	29
**91**	Molleblossomin K	*Rhododendron molle*, flowers	2024	29
**92**	Molleblossomin L	*Rhododendron molle*, flowers	2024	29
**93**	16-Acetylgrayanotoxin III	*Rhododendron micranthum*, roots	2020	19
**94**	3β, 6β, 16α-trihydroxy-14b-acetoxy-grayan-1(5), 10(20)-diene	*Rhododendron micranthum*, roots	2020	19
**95**	14β-(2-Hydroxypropanoyloxy)rhodomollein XVII	*Craibiodendron yunnanense*, leaves	2023	30
**96**	2-O-Ethoxyrhodojaponin VI	*Craibiodendron yunnanense*, leaves	2023	30
**97**	Micranthanoside J	*Craibiodendron yunnanense*, leaves	2023	30
**98**	Mollfoliagein A	*Rhododendron molle*, leaves	2018	7
**99**	Mollfoliagein B	*Rhododendron molle*, leaves	2018	7
**100**	Mollfoliagein C	*Rhododendron molle*, leaves	2018	7
**101**	6-O-Acetylrhodomollein XXXI	*Rhododendron molle*, leaves	2018	7
**102**	Mollfoliagein E	*Rhododendron molle*, leaves	2018	7
**103**	Rhododecorumin VI	*Rhododendron decorum*, leaves and twigs	2018	22
**104**	Rhododecorumin VII	*Rhododendron decorum*, leaves and twigs	2018	22
**105**	Epoxypieristoxin A	*Pieris formosa*, roots	2019	31
**106**	Epoxypieristoxin B	*Pieris formosa*, roots	2019	31
**107**	Epoxypieristoxin C	*Pieris formosa*, roots	2019	31
**108**	Epoxypieristoxin D	*Pieris formosa*, roots	2019	31

**109**	Epoxypieristoxin E	*Pieris formosa*, roots	2019	31
**110**	Epoxypieristoxin F	*Pieris formosa*, roots	2019	31
**111**	Epoxypieristoxin G	*Pieris formosa*, roots	2019	31
**112**	Epoxypieristoxin H	*Pieris formosa*, roots	2019	31
**113**	14-Deoxyrhodomollein XXXVII	*Pieris japonica*, leaves	2019	26
**114**	Rhodomollein XLVIII	*Rhododendron molle*, flowers	2020	20
**115**	Micranthanol A	*Rhododendron micranthum*, leaves	2021	17
**116**	Micranthanol B	*Rhododendron micranthum*, leaves	2021	17
**117**	Daublossomin A	*Rhododendron dauricum*, flowers	2023	27
**118**	Daublossomin B	*Rhododendron dauricum*, flowers	2023	27
**119**	Daublossomin C	*Rhododendron dauricum*, flowers	2023	27
**120**	Daublossomin D	*Rhododendron dauricum*, flowers	2023	27
**121**	Daublossomin E	*Rhododendron dauricum*, flowers	2023	27
**122**	Daublossomin F	*Rhododendron dauricum*, flowers	2023	27
**123**	Craibiodenoside D	*Craibiodendron yunnanense*, leaves	2023	28
**124**	Craibiodenoside E	*Craibiodendron yunnanense*, leaves	2023	28
**125**	Craibiodenoside F	*Craibiodendron yunnanense*, leaves	2023	28
**126**	Molleblossomin A	*Rhododendron molle*, flowers	2024	29
**127**	Molleblossomin B	*Rhododendron molle*, flowers	2024	29
**128**	Molleblossomin C	*Rhododendron molle*, flowers	2024	29
**129**	Molleblossomin D	*Rhododendron molle*, flowers	2024	29
**130**	Molleblossomin E	*Rhododendron molle*, flowers	2024	29
**131**	Molleblossomin F	*Rhododendron molle*, flowers	2024	29
**132**	Auriculatol A	*Rhododendron auriculatum*, leaves	2019	25
**133**	9β-Hydroxy-1,5-seco-grayanotoxin	*Rhododendron micranthum*, leaves	2021	17
**134**	Dauricanol D	*Rhododendron dauricum*, flowers	2023	16
**135**	Dauricanol E	*Rhododendron dauricum*, flowers	2023	16
**136**	Pierisjaponin A	*Pieris japonica*, leaves	2020	18
**137**	Pierisjaponin B	*Pieris japonica*, leaves	2020	18
**138**	Rhodoauriculatol A	*Rhododendron auriculatum*, leaves	2019	21
**139**	Rhodoauriculatol B	*Rhododendron auriculatum*, leaves	2019	21
**140**	Rhodoauriculatol C	*Rhododendron auriculatum*, leaves	2019	21
**141**	Rhodoauriculatol D	*Rhododendron auriculatum*, leaves	2019	21
**142**	Pierisjaponin J	*Pieris japonica*, leaves	2020	18
**143**	Birhodomollein D	*Rhododendron pumilum*, fruits	2018	32
**144**	Birhodomollein E	*Rhododendron pumilum*, fruits	2018	32
**145**	Bimollfoliagein A	*Rhododendron molle*, leaves	2018	7
**146**	Rhodomollein XLIII	*Rhododendron molle*, flowers	2020	20
**147**	Bismollether A	*Rhododendron molle*, flowers	2022	24
**148**	Bismollether B	*Rhododendron molle*, flowers	2022	24
**149**	Bismollether C	*Rhododendron molle*, flowers	2022	24
**150**	Rhododecorumin I	*Rhododendron decorum*, leaves and twigs	2018	22
**151**	Rhododecorumin II	*Rhododendron decorum*, leaves and twigs	2018	22
**152**	Rhododecorumin III	*Rhododendron decorum*, leaves and twigs	2018	22
**153**	Rhodoauriculatol G	*Rhododendron auriculatum*, leaves	2019	21
**154**	Rhodoauriculatol H	*Rhododendron auriculatum*, leaves	2019	21
**155**	Rhodomicranoside A	*Rhododendron auriculatum*, leaves	2019	14
**156**	Rhodomicranoside B	*Rhododendron auriculatum*, leaves	2019	14
**157**	Rhodomicranoside C	*Rhododendron auriculatum*, leaves	2019	14
**158**	Rhodomollein LII	*Rhododendron molle*, flowers	2020	20
**159**	Rhodomollein LIII	*Rhododendron molle*, flowers	2020	20
**160**	3β,7α,14β-trihydroxy-leucoth-10(20),15-dien-5-one	*Pieris formosa*, roots	2020	15
**161**	10α,16α-dihydroxy-leucoth-5-one	*Pieris formosa*, roots	2020	15
**162**	Pierisjaponin F	*Pieris japonica*, leaves	2020	18
**163**	Pierisjaponin G	*Pieris japonica*, leaves	2020	28
**164**	Rhodoauriculatol F	*Rhododendron auriculatum*, leaves	2019	21

**165**	Pierisentkauran B	*Pieris formosa*, roots	2020	15
**166**	Pierisentkauran C	*Pieris formosa*, roots	2020	15
**167**	Pierisentkauran D	*Pieris formosa*, roots	2020	15
**168**	Pierisentkauran E	*Pieris formosa*, roots	2020	15
**169**	Rhodomicranoside D	*Rhododendron micranthum*, leaves	2019	14
**170**	Rhodomicranoside E	*Rhododendron micranthum*, leaves	2019	14
**171**	Pierisentkauran F	*Pieris formosa*, roots	2020	15
**172**	Pierisjaponin H	*Pieris japonica*, leaves	2020	18
**173**	Pierisjaponin I	*Pieris japonica*, leaves	2020	18
**174**	8α-O-Acetylrhodomollein XXIII	*Rhododendron micranthum*, leaves	2021	17
**175**	Rhodokalmanol A	*Rhododendron dauricum*, leaves	2022	33
**176**	Rhodokalmanol B	*Rhododendron dauricum*, leaves	2022	33
**177**	Rhodokalmanol C	*Rhododendron dauricum*, leaves	2022	33
**178**	Rhodokalmanol D	*Rhododendron dauricum*, leaves	2022	33
**179**	16α-acetoxy rhodomollein XXIII	*Rhododendron micranthum*, roots	2020	19
**180**	Rhodomollein LI	*Rhododendron molle*, flowers	2020	20
**181**	Rhodoauriculatol E	*Rhododendron auriculatum*, leaves	2019	21
**182**	Mollebenzylanol A	*Rhododendron molle*, leaves	2018	23
**183**	Mollebenzylanol B	*Rhododendron molle*, leaves	2018	23
**184**	Rhododecorumin IV	*Rhododendron decorum*, leaves and twigs	2018	22
**185**	Rhododecorumin V	*Rhododendron decorum*, leaves and twigs	2018	22
**186**	Micranthanone B	*Rhododendron micranthum*, leaves	2021	17
**187**	Micranthanone C	*Rhododendron micranthum*, leaves	2021	17
**188**	14-epi-Mollanol A	*Rhododendron micranthum*, leaves	2021	17
**189**	Mollanol B	*Rhododendron micranthum*, leaves	2021	17
**190**	Mollanol C	*Rhododendron micranthum*, leaves	2021	17
**191**	Pierisjaponin E	*Pieris japonica*, leaves	2020	18
**192**	Rhomollone A	*Rhododendron molle*, flowers	2020	20
**193**	rhodauricanol A	*Rhododendron dauricum*, flowers	2023	16

Table S2. Compound names and reported activities.

no	name	Test model/ in vivo	Activity/ dose	Test model/in vitro	Activity/ dose
**1**	Pierisformosoid A	Acetic acid-Induced pain mouse model Plutella xylostella	Analgesic, 5 mg/kg<br>Antifeedant, 0.5 mg/mL	Nav1.7 channel<br>KCNQ2 channel	ND, 10 µM<br>ND, 10 µM
**2**	Pierisformosoid B	Acetic acid-Induced pain mouse model	Analgesic, 1 mg/kg	Nav1.7 channel<br>KCNQ2 channel	ND, 10 µM<br>ND, 10 µM
**3**	Pierisformosoid C	-	-	Nav1.7 channel<br>KCNQ2 channel	ND, 10 µM<br>ND, 10 µM
**4**	Pierisformosoid D	Acetic acid-induced pain mouse model Plutella xylostella	Analgesic, 0.1 mg/kg<br>Antifeedant, 0.5 mg/mL	Nav1.7 channel<br>KCNQ2 channel	ND, 10 µM<br>38.3% inhibitory, 10 µM
**5**	Pierisformosoid E	Acetic acid-Induced pain mouse model	Analgesic, 5 mg/kg	Nav1.7 channel<br>KCNQ2 channel	ND, 10 µM<br>ND, 10 µM
**6**	Pierisformosoid F	-	-	Nav1.7 channel<br>KCNQ2 channel	ND, 10 µM<br>ND, 10 µM

**7**	Pierisformosoid G	Acetic acid-Induced pain mouse model	Analgesic, 0.1 mg/kg	Nav1.7 channel<br>KCNQ2 channel	ND, 10 µM<br>ND, 10 µM
**8**	Pierisformosoid H	Acetic acid-Induced pain mouse model	Analgesic, 5 mg/kg	Nav1.7 channel<br>KCNQ2 channel	ND, 10 µM<br>ND, 10 µM
**9**	Pierisformosoid I	Acetic acid-Induced pain mouse model Plutella xylostella	ND<br>Antifeedant, 0.5 mg/mL	Nav1.7 channel<br>KCNQ2 channel	ND, 10 µM<br>ND, 10 µM
**10**	Pierisformosoid J	Acetic acid-Induced pain mouse model	ND	Nav1.7 channel<br>KCNQ2 channel	ND, 10 µM<br>ND, 10 µM
**11**	Pierisformosoid K	Acetic acid-Induced pain mouse model	ND	Nav1.7 channel<br>KCNQ2 channel	ND, 10 µM<br>ND, 10 µM
**12**	Pierisformosoid L	Acetic acid-Induced pain mouse model	ND	Nav1.7 channel<br>KCNQ2 channel	ND, 10 µM<br>ND, 10 µM
**13**	3-epi-grayanoside B	Acetic acid-Induced pain mouse model	Analgesic, 5.0 mg/kg	Anti-inflammatory<br>Cytotoxicity<br>PTP1B	ND, 40 µM<br>ND, 40 µM<br>ND, 40 µM
**14**	Micranthanoside A	Acetic acid-Induced pain mouse model	Analgesic, 0.2 mg/kg	Anti-inflammatory<br>Cytotoxicity<br>PTP1B	ND, 40 µM<br>ND, 40 µM<br>ND, 40 µM
**15**	Micranthanoside B	Acetic acid-Induced pain mouse model	Analgesic, 1.0 mg/kg	Anti-inflammatory<br>Cytotoxicity<br>PTP1B	ND, 40 µM<br>ND, 40 µM<br>ND, 40 µM
**16**	Micranthanoside C	Acetic acid-Induced pain mouse model	Analgesic, 5.0 mg/kg	Anti-inflammatory<br>Cytotoxicity<br>PTP1B	ND, 40 µM<br>ND, 40 µM<br>ND, 40 µM
**17**	Micranthanoside D	Acetic acid-Induced pain mouse model	Analgesic, 5.0 mg/kg	Anti-inflammatory<br>Cytotoxicity<br>PTP1B	ND, 40 µM<br>ND, 40 µM<br>ND, 40 µM
**18**	Micranthanoside E	Acetic acid-Induced pain mouse model	Analgesic, 5.0 mg/kg	Anti-inflammatory<br>Cytotoxicity<br>PTP1B	ND, 40 µM<br>ND, 40 µM<br>ND, 40 µM
**19**	hydroxygrayanoside C	Acetic acid-Induced pain mouse model	Analgesic, 1.0 mg/kg	Anti-inflammatory<br>Cytotoxicity<br>PTP1B	ND, 40 µM<br>ND, 40 µM<br>ND, 40 µM
**20**	micranthanoside F	Acetic acid-Induced pain mouse model	Analgesic, 5.0 mg/kg	Anti-inflammatory<br>Cytotoxicity<br>PTP1B	ND, 40 µM<br>ND, 40 µM<br>ND, 40 µM
**21**	14β-acetyoxymicranthanoside	Acetic acid-Induced pain mouse model	Analgesic, 5.0 mg/kg	Anti-inflammatory<br>Cytotoxicity<br>PTP1B	ND, 40 µM<br>ND, 40 µM<br>ND, 40 µM
**22**	micranthanoside G	Acetic acid-Induced pain mouse model	Analgesic, 5.0 mg/kg	Anti-inflammatory<br>Cytotoxicity<br>PTP1B	ND, 40 µM<br>ND, 40 µM<br>ND, 40 µM
**23**	14-Oacetylmicranthanoside G	Acetic acid-Induced pain mouse model	Analgesic, 5.0 mg/kg	Anti-inflammatory<br>Cytotoxicity<br>PTP1B	ND, 40 µM<br>ND, 40 µM<br>ND, 40 µM
**24**	14β-hydroxypieroside A	Acetic acid-Induced pain mouse model	Analgesic, 5.0 mg/kg	Anti-inflammatory<br>Cytotoxicity<br>PTP1B	ND, 40 µM<br>ND, 40 µM<br>ND, 40 µM
**25**	micranthanoside H	Acetic acid-Induced pain mouse model	Analgesic, 5.0 mg/kg	Anti-inflammatory<br>Cytotoxicity<br>PTP1B	ND, 40 µM<br>ND, 40 µM<br>ND, 40 µM

**26**	Mollfoliagein D	-		Anti-inflammatory	ND, 40 μM
**27**	6-O-Acetylrhodomollein XI	-		Anti-inflammatory	ND, 40 μM
**28**	Mollfoliagein F	--		Anti-inflammatory	ND, 40 μM
**29**	18-Hydroxygrayanotoxin XVIII			Anti-inflammatory	ND, 40 μM
**30**	2-O-Methylrhodomolin I	-		Anti-inflammatory	ND, 40 μM
**31**	2-O-Methylrhodomollein XII	-		Anti-inflammatory	ND, 40 μM
**32**	2-O-Methylrhodojaponin VI	-		Anti-inflammatory	ND, 40 μM
**33**	2-O-Methylrhodojaponin VII	-		Anti-inflammatory	ND, 40 μM
**34**	Rhododecorumin VIII	Acetic acid-Induced pain mouse model	Analgesic, 10.0 mg/kg	-	
**35**	Rhododecorumin IX	-		-	
**36**	Rhododecorumin X	Acetic acid-Induced pain mouse model	Analgesic, 10.0 mg/kg	-	
**37**	Rhododecorumin XI	Acetic acid-Induced pain mouse model	Analgesic, 10.0 mg/kg	-	
**38**	Rhododecorumin XII	Acetic acid-induced pain mouse model	Analgesic, 0.8 mg/kg	-	
**39**	Rhododeoside I	Acetic acid-Induced pain mouse model	Analgesic, 10.0 mg/kg	-	
**40**	Rhodoauriculatol I	Acetic acid-Induced pain mouse model	Analgesic, 1.0 mg/kg	-	
**41**	Rhodomicranoside F	Acetic acid-Induced pain mouse model	Analgesic, 5.0 mg/kg	-	
**42**	Rhodomicranoside G	Acetic acid-Induced pain mouse model	Analgesic, 5.0 mg/kg	-	
**43**	Rhodomicranoside H	Acetic acid-Induced pain mouse model	Analgesic, 1.0 mg/kg	-	
**44**	Rhodomicranoside I	Acetic acid-Induced pain mouse model	Analgesic, 1.0 mg/kg	-	
**45**	Auriculatol B	Acetic acid-Induced pain mouse model	Analgesic, 5.0 mg/kg	-	
**46**	3-epi-Grayanotoxin XVIII	Acetic acid-Induced pain mouse model	Analgesic, 5.0 mg/kg	-	
**47**	6-Deoxycraiobiotoxin I	Acetic acid-Induced pain mouse model	Analgesic, 5.0 mg/kg	-	
**48**	3-epi-Auriculatol B	Acetic acid-Induced pain mouse model	Analgesic, 5.0 mg/kg	-	
**49**	19-Hydroxy-3-epi-auriculatol B	Acetic acid-Induced pain mouse model	Analgesic, 5.0 mg/kg	-	
**50**	Auriculatol C	Acetic acid-Induced pain mouse model	Analgesic, 5.0 mg/kg	-	
**51**	Auriculatol D	Acetic acid-Induced pain mouse model	Analgesic, 5.0 mg/kg	-	
**52**	Auriculatol E	Acetic acid-Induced pain mouse model	Analgesic, 1.0 mg/kg	-	
**53**	Auriculatol F	Acetic acid-Induced pain mouse model	Analgesic, 5.0 mg/kg	-	
**54**	2α-Hydroxyauriculatol F	Acetic acid-Induced pain mouse model	Analgesic, 5.0 mg/kg	-	
**55**	1-epi-Pieristoxin S	Acetic acid-Induced pain mouse model	Analgesic, 5.0 mg/kg	-	
**56**	17-Hydroxygrayanotoxin XIX	Acetic acid-Induced pain mouse model	Analgesic, 0.04 mg/kg	-	
**57**	2-O-Methylrhodomollein XIX	Acetic acid-Induced pain mouse model	Analgesic, 5.0 mg/kg	-	

**58**	17-Hydroxy-3-epi-auriculatol B	Acetic acid-Induced pain mouse model	Analgesic, 5.0 mg/kg	-	
**59**	Pierisjaponol A	Acetic acid-Induced pain mouse model	Analgesic, 0.2 mg/kg	-	
**60**	Pierisjaponol B	Acetic acid-Induced pain mouse model	Analgesic, 5.0 mg/kg	-	
**61**	13α-Hydroxyrhodomollein XVII	Acetic acid-Induced pain mouse model	Analgesic, 5.0 mg/kg	-	
**62**	12β-Hydroxygrayanotoxin XVIII	Acetic acid-Induced pain mouse model	Analgesic, 5.0 mg/kg	-	
**63**	2α-Hydroxyasebotoxin II	Acetic acid-Induced pain mouse model	Analgesic, 5.0 mg/kg	-	
**64**	2α-O-Methylgrayanotoxin II	Acetic acid-Induced pain mouse model	Analgesic, 5.0 mg/kg	-	
**65**	Pierisjaponol C	Acetic acid-Induced pain mouse model	Analgesic, 5.0 mg/kg	-	
**66**	16-O-Methylgrayanotoxin XVIII	Acetic acid-Induced pain mouse model	Analgesic, 5.0 mg/kg	-	
**67**	Pierisjaponol D	Acetic acid-Induced pain mouse model	Analgesic, 5.0 mg/kg	-	
**68**	Rhodomollein XLIV	Acetic acid-Induced pain mouse model	Analgesic, 20.0 mg/kg	-	
**69**	Rhodomollein XLV	Acetic acid-Induced pain mouse model	Analgesic, 20.0 mg/kg	-	
**70**	Rhodomollein XLVI	-		-	
**71**	Rhodomollein XLVII	Acetic acid-Induced pain mouse model	Analgesic, 2.0 mg/kg	-	
**72**	Rhodomollein XLIX	-		-	
**73**	Rhodomollein L	-		-	
**74**	Dauricanol A	Acetic acid-Induced pain mouse model	Analgesic, 5.0 mg/kg		
**75**	Dauricanol B	Acetic acid-Induced pain mouse model	Analgesic, 0.04 mg/kg		
**76**	Dauricanol C	Acetic acid-Induced pain mouse model	Analgesic, 0.04 mg/kg		
**77**	Daublossomin G	Acetic acid-Induced pain mouse model	Analgesic, 5.0 mg/kg		
**78**	Daublossomin H	Acetic acid-Induced pain mouse model	Analgesic, 5.0 mg/kg		
**79**	Daublossomin I	Acetic acid-Induced pain mouse model	ND		
**80**	Daublossomin J	Acetic acid-Induced pain mouse model	Analgesic, 5.0 mg/kg		
**81**	Daublossomin K	Acetic acid-Induced pain mouse model	Analgesic, 5.0 mg/kg		
**82**	Daublossomin L	-			
**83**	Daublossomin M	Acetic acid-Induced pain mouse model	Analgesic, 5.0 mg/kg		
**84**	Craibiodenoside A			Anti-inflammatory	ND, 10 μg/mL
**85**	Craibiodenoside B			Anti-inflammatory	10 μg/mL
**86**	Craibiodenoside C			Anti-inflammatory	10 μg/mL
**87**	Molleblossomin G	Acetic acid-Induced pain mouse model	Analgesic, 5.0 mg/kg	-	
**88**	Molleblossomin H	Acetic acid-Induced pain mouse model	Analgesic, 5.0 mg/kg	-	
**89**	Molleblossomin I	Acetic acid-Induced pain mouse model	Analgesic, 5.0 mg/kg	-	
**90**	Molleblossomin J	Acetic acid-Induced pain mouse model	Analgesic, 5.0 mg/kg	-	
**91**	Molleblossomin K	Acetic acid-Induced pain mouse model	Analgesic, 5.0 mg/kg	-	

**92**	Molleblossomin L	Acetic acid-Induced pain mouse model	Analgesic, 5.0 mg/kg	-	
**93**	16-Acetylgrayanotoxin III	Acetic acid-Induced pain mouse model	Analgesic, 1.0 mg/kg	-	
**94**	3β, 6β, 16α-trihydroxy-14b-acetoxy-grayan-<br>1(5), 10(20)-diene	Acetic acid-Induced pain mouse model	Analgesic, 0.8 mg/kg	-	
**95**	14β-(2-Hydroxypropanoyloxy)rhodomollein XVII	-		-	
**96**	2-O-Ethoxyrhodojaponin VI	-		-	
**97**	Micranthanoside J	-		-	
**98**	Mollfoliagein A	-		Anti-inflammatory	ND, 40 µM
**99**	Mollfoliagein B	-		Anti-inflammatory	ND, 40 µM
**100**	Mollfoliagein C	-		Anti-inflammatory	IC_{50} 35.4 ± 3.9 µM
**101**	6-O-Acetylrhodomollein XXXI	-		Anti-inflammatory	ND, 40 µM
**102**	Mollfoliagein E	-		Anti-inflammatory	ND, 40 µM
**103**	Rhododecorumin VI	Acetic acid-Induced pain mouse model	Analgesic, 10.0 mg/kg	-	
**104**	Rhododecorumin VII	-		-	
**105**	Epoxypieristoxin A	Acetic acid-Induced pain mouse model	Analgesic, 5.0 mg/kg	-	
**106**	Epoxypieristoxin B	Acetic acid-Induced pain mouse model	Analgesic, 5.0 mg/kg	-	
**107**	Epoxypieristoxin C	Acetic acid-Induced pain mouse model	Analgesic, 5.0 mg/kg	-	
**108**	Epoxypieristoxin D	-		-	
**109**	Epoxypieristoxin E	Acetic acid-Induced pain mouse model	Analgesic, 5.0 mg/kg	-	
**110**	Epoxypieristoxin F	Acetic acid-Induced pain mouse model	Analgesic, 5.0 mg/kg	-	
**111**	Epoxypieristoxin G	Acetic acid-Induced pain mouse model	Analgesic, 5.0 mg/kg	-	
**112**	Epoxypieristoxin H	Acetic acid-Induced pain mouse model	Analgesic, 5.0 mg/kg	-	
**113**	14-Deoxyrhodomollein XXXVII	Acetic acid-Induced pain mouse model	Analgesic, 5.0 mg/kg	-	
**114**	Rhodomollein XLVIII	Acetic acid-Induced pain mouse model	Analgesic, 20.0 mg/kg	-	
**115**	Micranthanol A	Acetic acid-Induced pain mouse model	Analgesic, 5.0 mg/kg	-	
**116**	Micranthanol B	Acetic acid-Induced pain mouse model	Analgesic, 5.0 mg/kg	-	
**117**	Daublossomin A	Acetic acid-Induced pain mouse model	Analgesic, 0.2 mg/kg	-	
**118**	Daublossomin B	Acetic acid-Induced pain mouse model	Analgesic, 5.0 mg/kg	-	
**119**	Daublossomin C	Acetic acid-Induced pain mouse model	Analgesic, 5.0 mg/kg	-	
**120**	Daublossomin D	Acetic acid-Induced pain mouse model	Analgesic, 5.0 mg/kg	-	
**121**	Daublossomin E	Acetic acid-Induced pain mouse model	Analgesic, 5.0 mg/kg	-	
**122**	Daublossomin F	Acetic acid-Induced pain mouse model	Analgesic, 0.2 mg/kg	-	
**123**	Craibiodenoside D	-		Anti-inflammatory	ND, 10 µg/mL
**124**	Craibiodenoside E	-		Anti-inflammatory	ND, 10 µg/mL

**125**	Craibiodenoside F	-		Anti-inflammatory	10 μg/mL
**126**	Molleblossomin A	Acetic acid-Induced pain mouse model	Analgesic, 5.0 mg/kg	-	
**127**	Molleblossomin B	Acetic acid-Induced pain mouse model	Analgesic, 5.0 mg/kg	-	
**128**	Molleblossomin C	Acetic acid-Induced pain mouse model	Analgesic, 5.0 mg/kg	-	
**129**	Molleblossomin D	Acetic acid-Induced pain mouse model	Analgesic, 5.0 mg/kg	-	
**130**	Molleblossomin E	Acetic acid-Induced pain mouse model	Analgesic, 5.0 mg/kg	-	
**131**	Molleblossomin F	Acetic acid-Induced pain mouse model	Analgesic, 0.2 mg/kg	-	
**132**	Auriculatol A	Acetic acid-Induced pain mouse model	Analgesic, 5.0 mg/kg	-	
**133**	9β-Hydroxy-1,5-seco-grayanotoxin	Acetic acid-Induced pain mouse model	Analgesic, 5.0 mg/kg	-	
**134**	Dauricanol D	Acetic acid-Induced pain mouse model	Analgesic, 1.0 mg/kg	-	
**135**	Dauricanol E	Acetic acid-Induced pain mouse model	Analgesic, 1.0 mg/kg	-	
**136**	Pierisjaponin A	Acetic acid-Induced pain mouse model	Analgesic, 1.0 mg/kg	-	
**137**	Pierisjaponin B	Acetic acid-Induced pain mouse model	Analgesic, 0.04 mg/kg	-	
**138**	Rhodoauriculatol A	Acetic acid-Induced pain mouse model	Analgesic, 5.0 mg/kg	-	
**139**	Rhodoauriculatol B	Acetic acid-Induced pain mouse model	Analgesic, 5.0 mg/kg	-	
**140**	Rhodoauriculatol C	Acetic acid-Induced pain mouse model	Analgesic, 5.0 mg/kg	-	
**141**	Rhodoauriculatol D	Acetic acid-Induced pain mouse model	Analgesic, 5.0 mg/kg	-	
**142**	Pierisjaponin J	Acetic acid-Induced pain mouse model	Analgesic, 5.0 mg/kg	-	
**143**	Birhodomollein D	-		-	
**144**	Birhodomollein E	-		-	
**145**	Bimollfoliagein A	-		Anti-inflammatory	ND, 40 μM
**146**	Rhodomollein XLIII	-		-	
**147**	Bismollether A	Acetic acid-Induced pain mouse model	Analgesic, 5.0 mg/kg	-	
**148**	Bismollether B	Acetic acid-Induced pain mouse model<br>Capsaicin-Induced pain mouse model<br>AITC-Induced pain mouse model	Analgesic, 0.2 mg/kg<br>Analgesic, 5.0 mg/kg<br>Analgesic, 5.0 mg/kg		
**149**	Bismollether C	Acetic acid-Induced pain mouse model	Analgesic, 5.0 mg/kg		
**150**	Rhododecorumin I	Acetic acid-Induced pain mouse model	Analgesic, 10.0 mg/kg	-	
**151**	Rhododecorumin II	-		-	
**152**	Rhododecorumin III	Acetic acid-Induced pain mouse model	Analgesic, 10.0 mg/kg	-	
**153**	Rhodoauriculatol G	Acetic acid-Induced pain mouse model	Analgesic, 5.0 mg/kg	-	
**154**	Rhodoauriculatol H	Acetic acid-Induced pain mouse model	Analgesic, 5.0 mg/kg	-	
**155**	Rhodomicranoside A	Acetic acid-Induced pain mouse model	Analgesic, 1.0 mg/kg	-	
**156**	Rhodomicranoside B	Acetic acid-Induced pain mouse model	Analgesic, 1.0 mg/kg	-	

**157**	Rhodomicranoside C	Acetic acid-Induced pain mouse model	Analgesic, 1.0 mg/kg	-	
**158**	Rhodomollein LII	-		-	
**159**	Rhodomollein LIII	Acetic acid-Induced pain mouse model	Analgesic, 5.0 mg/kg	-	
**160**	3β,7α,14β-trihydroxy-leucoth-10(20),15-dien-5-one	Acetic acid-Induced pain mouse model	Analgesic, 5.0 mg/kg	-	
**161**	10α,16α-dihydroxy-leucoth-5-one	Acetic acid-Induced pain mouse model	Analgesic, 5 mg/kg Antifeedant, 0.5 mg/mL	-	
**162**	Pierisjaponin F	Acetic acid-Induced pain mouse model	Analgesic, 1.0 mg/kg	-	
**163**	Pierisjaponin G	Acetic acid-Induced pain mouse model	Analgesic, 5.0 mg/kg	-	
**164**	Rhodoauriculatol F	Acetic acid-Induced pain mouse model	Analgesic, 5.0 mg/kg	-	
**165**	Pierisentkauran B	-		-	
**166**	Pierisentkauran C	Acetic acid-Induced pain mouse model	Analgesic, 5.0 mg/kg	-	
**167**	Pierisentkauran D	Plutella xylostella	Antifeedant, 0.5 mg/mL	-	
**168**	Pierisentkauran E	-		-	
**169**	Rhodomicranoside D	Acetic acid-Induced pain mouse model	Analgesic, 1.0 mg/kg	-	
**170**	Rhodomicranoside E	Acetic acid-Induced pain mouse model	Analgesic, 1.0 mg/kg	-	
**171**	Pierisentkauran F	Acetic acid-Induced pain mouse model	Analgesic, 5.0 mg/kg	-	
**172**	Pierisjaponin H	Acetic acid-Induced pain mouse model	Analgesic, 5.0 mg/kg	-	
**173**	Pierisjaponin I	Acetic acid-Induced pain mouse model	Analgesic, 1.0 mg/kg	-	
**174**	8α-O-Acetylrhodomollein XXIII	Acetic acid-Induced pain mouse model	Analgesic, 5.0 mg/kg	-	
**175**	Rhodokalmanol A	Acetic acid-Induced pain mouse model	Analgesic, 1.0 mg/kg	-	
**176**	Rhodokalmanol B	Acetic acid-Induced pain mouse model	Analgesic, 5.0 mg/kg	-	
**177**	Rhodokalmanol C	Acetic acid-Induced pain mouse model	Analgesic, 0.04 mg/kg	-	
**178**	Rhodokalmanol D	Acetic acid-Induced pain mouse model	Analgesic, 0.2 mg/kg	-	
**179**	16α-acetoxy rhodomollein XXIII	Acetic acid-Induced pain mouse model	Analgesic, 1.0 mg/kg	-	
**180**	Rhodomollein LI	-		-	
**181**	Rhodoauriculatol E	Acetic acid-Induced pain mouse model	Analgesic, 5.0 mg/kg	-	
**182**	Mollebenzylanol A	-		PTP1B	IC_{50} 22.99 ± 0.43 µM
**183**	Mollebenzylanol B	-		PTP1B	IC_{50} 32.24 ± 0.74 µM
**184**	Rhododecorumin IV	Acetic acid-Induced pain mouse model	Analgesic, 10.0 mg/kg	-	
**185**	Rhododecorumin V	-		-	
**186**	Micranthanone B	Acetic acid-Induced pain mouse model	Analgesic, 1.0 mg/kg		
**187**	Micranthanone C	Acetic acid-Induced pain mouse model	Analgesic, 1.0 mg/kg		
**188**	14-epi-Mollanol A	Acetic acid-Induced pain mouse model	Analgesic, 5.0 mg/kg		
**189**	Mollanol B	Acetic acid-Induced pain mouse model	Analgesic, 5.0 mg/kg		

**190**	Mollanol C	Acetic acid-Induced pain mouse model	Analgesic, 5.0 mg/kg		
**191**	Pierisjaponin E	Acetic acid-Induced pain mouse model	Analgesic, 5.0 mg/kg	-	
**192**	Rhomollone A	-		-	
**193**	Rhodauricanol A	Acetic acid-Induced pain mouse model	Analgesic, 0.2 mg/kg	-	

ND: Activity not detected;   -: Didn't report in the literature.
